# Sex-related differences in serum biomarker levels predict the activity and efficacy of immune checkpoint inhibitors in advanced melanoma and non-small cell lung cancer patients

**DOI:** 10.1186/s12967-024-04920-6

**Published:** 2024-03-05

**Authors:** Giulia Pasello, Aline S. C. Fabricio, Paola Del Bianco, Valentina Salizzato, Adolfo Favaretto, Luisa Piccin, Fable Zustovich, Alessio Fabozzi, Costanza De Rossi, Jacopo Pigozzo, Mattia De Nuzzo, Elia Cappelletto, Laura Bonanno, Dario Palleschi, Gian Luca De Salvo, Valentina Guarneri, Massimo Gion, Vanna Chiarion-Sileni

**Affiliations:** 1 Medical Oncology 2, Veneto Institute of Oncology IOV-IRCCS, Padua, Italy; 2 Department of Surgery, Oncology and Gastroenterology, University of Padova, Padua, Italy; 3 Clinical Research Unit, Veneto Institute of Oncology IOV-IRCCS, Padua, Italy; 4 Medical Oncology Unit, Ca’ Foncello Hospital, AULSS 2, Treviso, Italy; 5 Medical Oncology Unit, AULSS 1 Dolomiti, Belluno, Italy; 6 Medical Oncology 3, Veneto Institute of Oncology IOV-IRCCS, Padua, Italy; 7 Medical Oncology Unit, AULSS 3 Serenissima, Mestre-Venice, Italy; 8 Regional Center for Biomarkers, Department of Clinical Pathology, AULSS3 Serenissima, Venice, Italy

**Keywords:** ICIs, Predictive biomarkers, Cytokines, Melanoma, NSCLC, Precision medicine

## Abstract

**Background:**

Immune Checkpoint Inhibitors (ICIs) lead to durable response and a significant increase in long-term survival in patients with advanced malignant melanoma (MM) and Non-Small Cell Lung Cancer (NSCLC). The identification of serum cytokines that can predict their activity and efficacy, and their sex interaction, could improve treatment personalization.

**Methods:**

In this prospective study, we enrolled immunotherapy-naïve patients affected by advanced MM and NSCLC treated with ICIs. The primary endpoint was to dissect the potential sex correlations between serum cytokines (IL-1β, IL-2, IL-4, IL-5, IL-6, IL-8, IL-10, GM-CSF, MCP-1, TNF-ɑ, IP-10, VEGF, sPD-L1) and the objective response rate (ORR). Secondly, we analyzed biomarker changes during treatment related to ORR, disease control rate (DCR), progression free survival (PFS) and overall survival (OS). Blood samples, collected at baseline and during treatment until disease progression (PD) or up to 2 years, were analyzed using Luminex xMAP or ELLA technologies.

**Results:**

Serum samples from 161 patients (98 males/63 females; 92 MM/69 NSCLC) were analyzed for treatment response. At baseline, IL-6 was significantly lower in females (F) *versus* males (M); lower levels of IL-4 in F and of IL-6 in both sexes significantly correlated with a better ORR, while higher IL-4 and TNF-ɑ values were predictive of a lower ORR in F *versus* M. One hundred and sixty-five patients were evaluable for survival analysis: at multiple Cox regression, an increased risk of PD was observed in F with higher baseline values of IL-4, sPD-L1 and IL-10, while higher IL-6 was a negative predictor in males. In males, higher levels of GM-CSF predict a longer survival, whereas higher IL-1β predicts a shorter survival. Regardless of sex, high baseline IL-8 values were associated with an increased risk of both PD and death, and high IL-6 levels only with shorter OS.

**Conclusions:**

Serum IL-1β, IL-4, IL-6, IL-10, GM-CSF, TNF-ɑ, and sPD-L1 had a significant sex-related predictive impact on ORR, PFS and OS in melanoma and NSCLC patients treated with ICIs. These results will potentially pave the way for new ICI combinations, designed according to baseline and early changes of these cytokines and stratified by sex.

**Supplementary Information:**

The online version contains supplementary material available at 10.1186/s12967-024-04920-6.

## Background

Anti-PD-1/PD-L1 and anti CTLA-4 immune checkpoint inhibitors (ICIs) have led to a paradigm shift in the treatment of many solid tumors. Malignant Melanoma (MM) and Non-Small Cell Lung Cancer (NSCLC) are considered as forerunners in the clinical application of these innovative drugs, with remarkable improvement in patient survival [1–13].

The huge revolution and the greatest difference of ICIs with respect to other anticancer agents was that it provided durable responses and a significant increase in long-term survival [14]. Response to ICIs is difficult to predict, since it could occur late or after a pseudoprogression (an increase ≥ 20% in the size of target tumor lesions or appearance of new lesions not confirmed as progressive disease on subsequent imaging assessments) [15]; in other cases, despite the fact that the best response observed is only a disease stabilization, it could be long lasting and translate into a survival benefit [16]. Other patients could face hyperprogression, characterized by a rapid progression of the tumor after the initiation of ICIs [17], with higher risk of early death within the first 12 weeks of treatment. Indeed, the early identification of patients who could benefit most from ICI treatment is still an unmet medical need in oncology. Currently, the only biomarker driving treatment selection is PD-L1 expression, approved in Italy by the Italian Medicines Agency (*Agenzia Italiana del Farmaco,* AIFA) for patients with NSCLC and melanoma. However, immunohistochemistry levels of PD-L1 expression on tumor cells are insufficient to predict response to and survival outcome of ICI therapy [18, 19]*.* Other biomarkers predictive for response, survival and toxicity of ICI treatment have been investigated, including tumor tissue biomarkers (i.e. tumor mutational burden and MHC molecule expression), circulating immune cell biomarkers (i.e. CD4 + T-cells, myeloid-derived suppressor cells) and soluble systemic immune/inflammatory biomarkers (i.e. lactate dehydrogenase, C-reactive protein and cytokines).

Acquired resistance to ICIs is a dynamic process and the longitudinal biomarker changes in monitoring during the first cycles of treatment seem to be promising for early identification of refractory patients, thus deserving further investigation [20].

Well-recognized sex differences in immune response, autoimmune diseases, tumor incidence and outcome [21] have led to the analysis of the impact of sex in the efficacy of ICIs with respect to standard therapies, revealing a significantly greater efficacy in men with melanoma and NSCLC [22], even though controversial evidence emerged from recent meta-analyses [23–28]. Numerous studies investigating efficacy and safety biomarkers in patients treated with ICIs have been undertaken, however, definite conclusions and reliable predictive tools are lacking. Starting from this caveat and from the evidence on sex differences in ICI efficacy [29], we planned the present study in order to identify and monitor sex-related predictive circulating biomarkers of activity and efficacy during ICI therapy in melanoma and NSCLC patients.

## Patients and methods

### Study design and participants

**Fig. 1:**
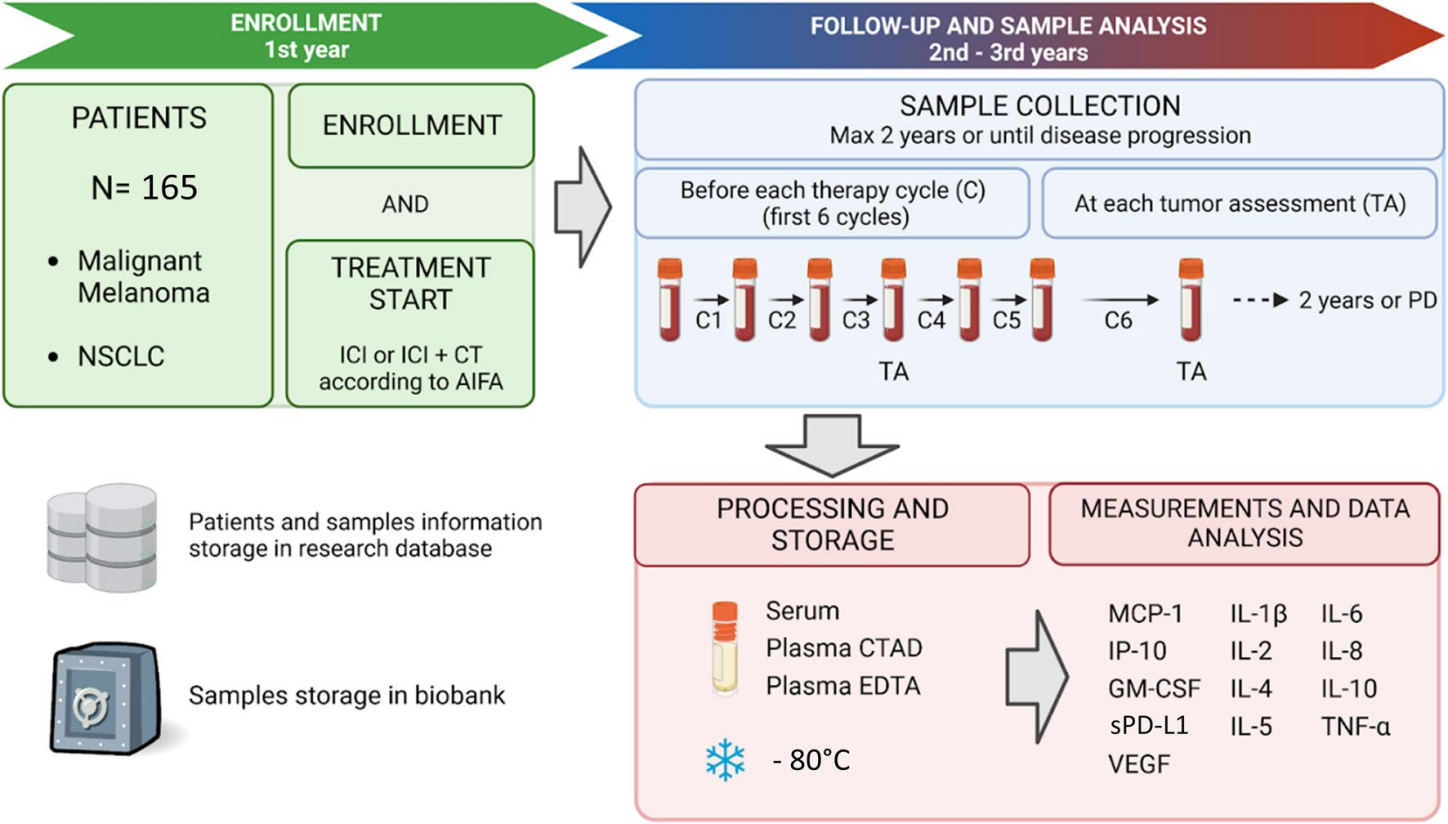
Study design. *ICI* immune checkpoint inhibitor, *CT* chemotherapy, *AIFA* Italian Medicines Agency, *TA* tumor assessment, *PD* progression disease, *C* therapy cycle, *CTAD* sodium citrate, theophylline, adenosine and dipyridamole, *EDTA* ethylenediamine tetraacetic acid. Created with BioRender.com

This is a prospective observational translational multicenter study enrolling MM and NSCLC patients eligible for treatment with ICIs in real-world clinical practice (Fig. 1), according to Italian regulatory approvals (Additional file 2: Table S1).

The primary endpoint of the study was to investigate the predictive role in terms of the objective response rate (ORR) to ICIs of 13 baseline inflammatory/immune-related circulating biomarkers and their potential sex interactions. Secondarily, we aimed to correlate serum cytokine behavior during treatment with ORR, disease control rate (DCR), progression free survival (PFS) and overall survival (OS).

The detection of adverse events and immune-related adverse events, differences between males and females and potential correlation with cytokine levels as early predictive markers of toxicity were secondary endpoints, and will be the object of a future publication.

The main eligibility criteria were: histologically confirmed diagnosis of unresectable stage III or IV MM or NSCLC (according to the VIII edition of the American Joint Committee on Cancer Staging Systems), receiving ICIs as first (or further) line treatment for advanced/metastatic disease, without symptomatic brain metastases or meningeal carcinomatosis, with an Eastern Cooperative Oncology Group (ECOG) Performance Status (PS) of 0–2. NSCLC patients with EGFR mutant or ALK rearranged tumors were not eligible.

All eligible patients, referred to four participating centers between April 2020 and July 2022, were evaluated for the study, and provided a signed informed consent form approved by the ethical committees prior to enrollment.

The patients received treatment with ICIs according to the clinical practice and regulatory approval criteria in Italy: nivolumab (Opdivo®) 240 or 480 mg flat dose every 2 or 4 weeks, respectively; pembrolizumab (Keytruda®) 200 mg flat dose every 3 weeks or atezolizumab (Tecentriq®) 1200 mg flat dose every 3 weeks. Treatment was administered until disease progression (PD), unacceptable toxicity or, in chemo-naïve NSCLC patients, for 35 cycles or 2 years of treatment. Details on the eligibility criteria, according to AIFA, are reported in Additional file 2: Table S1.

Radiological assessment with CT-scan was planned every 3 months according to the timeline defined by AIFA using the Response Evaluation Criteria in Solid Tumors (RECIST) version 1.1 for response evaluation.

### Collection, processing and storage of serum samples

All patients underwent blood sample collection at baseline, before each cycle of ICI therapy for the first 6 cycles and at every radiological assessment until confirmed PD or for a maximum of 2 years. Whole blood samples were collected using standard venipuncture techniques. In brief, using a Vacutainer adapter and double ended needle, blood was collected into one BD Vacutainer® Serum Separator Tube (SST^TM^ II Advance) (Becton, Dickinson and Company, Franklin Lakes, NJ, USA; 8.5 ml, yellow cap, with micronized silica as clot activator and gel separator). The serum tube was left to sit upright in a rack at room temperature for 30–45 min for clot formation to occur. Samples were then centrifuged at + 4 °C for 15 min at 2500 g and the supernatant (serum) immediately removed. Aliquots (500 μl) were immediately frozen, stored at − 80 °C at each site and sent to the IOV Biobank within 2 months of collection for storage until biomarker measurement (or long-term storage of residual material). Additional details about blood sample collection and processing are given in the Additional file 1: Supplementary Methods and elsewhere [30].

### Biomarker selection and measurement

The conceptual-methodological framework followed to select the immune-related biomarkers measured in the present study is reported in the Additional file 1: Supplementary Methods and elsewhere [30]. In brief, 28 immune-related biomarkers (interleukin (IL)-1⍺, IL-1β, IL-1 receptor antagonist (IL-1ra), IL-2, IL-4, IL-5, IL-6, IL-8, IL-10, IL-12 (p70), IL-13, IL-17A, epidermal growth factor (EGF), fibroblast growth factor (FGF)-2, granulocyte-colony stimulating factor (G-CSF), granulocyte macrophage colony stimulating factor (GM-CSF), growth-regulated protein (GRO)-⍺, interferon (IFN)-⍺, IFN-γ, interferon-inducible protein (IP-10), monocyte chemotactic protein (MCP-1), macrophage inflammatory protein (MIP)-1⍺, MIP-1β, soluble programmed death ligand-1 (sPD-L1), tumor necrosis factor (TNF)-⍺, vascular endothelial growth factor (VEGF), eotaxin and fractalkine were subjected to screening in 38 samples collected at baseline and during immunotherapy from 10 advanced melanoma or NSCLC patients, at different time points. Patient and sample characteristics are described elsewhere [30]. Twenty-five of the screened markers were found to be measurable in at least 30% of the tested serum samples, including IL-1β, IL-1ra, IL-2, IL-4, IL-5, IL-6, IL-8, IL-10, IL-12(p70), IL-13, IL-17A, EGF, G-CSF, GRO-α factor, GM-CSF, IFN-α, IFN-γ, IP-10, MCP-1, MIP-1β, sPD-L1, TNF-α, VEGF, eotaxin, fractalkine.

In a next step, taking into consideration the scientific evidence available and the availability of the molecules in customizable panels with adequate sensitivity, 13 markers were selected to be assayed in the samples of the entire series, including IL-1β, IL-2, IL-4, IL-5, IL-6, IL-8, IL-10, GM-CSF, TNF-⍺, MCP-1, IP-10, VEGF, sPD-L1.

For biomarker measurement, the samples were analyzed in the centralized laboratory, where they were freeze-thawed only once before assay. On the day of analysis, serum samples were quickly thawed in a 37 °C water bath and centrifuged at + 4 °C for 10 min at 16000 g. All serial samples, from the same patient, were measured in the same assay to reduce the effect of interassay variation on biomarker levels.

Immune-related biomarker levels were quantified in serum samples (upon 1:2 dilution) following the manufacturer’s protocol using a High Sensitivity Cytokine Premixed Kit A for 9 analytes (IL-1β, IL-2, IL-4, IL-5, IL-6, IL-8, IL-10, GM-CSF, TNF-⍺; Bio-Techne, CA, USA) on Bio-plex 200 system (Bio-Rad Laboratories Hercules, California, USA; xMAP multiplexing technology, Luminex) or a Simple-Plex cartridge for 4 analytes (MCP-1, IP-10, VEGF, sPD-L1; Bio-Techne, CA, USA) on ELLA Automated Immunoassay System Instrument (Bio-Techne, California, USA). Additional details about biomarker assessment are given in the Additional file 1: Supplementary Methods.

Quality control samples were generated by pooling serum, collected from multiple patients, which had measurable levels of the different cytokines as screened in the first phase of study. Two positive control samples with different levels of the target cytokines were analyzed on each run. All samples were analyzed in duplicate and the researcher was blinded to the outcome information.

### Statistical analysis and sample size calculation

In order to estimate an odds ratio of 0.1 for the interaction between sex and changes in biomarker levels with a study power of 80%, we established a significance level of 5% (2-sided), with an expected ORR of 35%, and an enrollment of 160 patients with a prevalence of 60% males.

The continuous variables were described as median and interquartile range (IQR) and their distributions according to different groups were compared using the Kruskal–Wallis test. Categorical variables were described as counts and percentages and compared between groups using the χ2 or Fisher exact test, as appropriate.

The clinical outcomes were analyzed in terms of ORR, DCR, PFS and OS.

ORR was defined as the achievement of a complete response (CR) or partial response (PR) according to RECIST 1.1 criteria. DCR was defined as the achievement of CR, PR or stable disease (SD). PFS was the time from the date of enrollment to the occurrence of PD or death from any cause. OS was the time from the date of enrollment to death from any cause. Patients who did not develop a survival event during the study period were censored at the date of last observation.

The nonparametric Kaplan–Meier method was used to estimate the survival probabilities and the median time from the Kaplan–Meier curve was provided along with the corresponding 95% confidence interval (CI) estimated using the Brookmeyer-Crowley method.

Each marker was analyzed for association with clinical outcome as a categorical variable according to high and low levels. Optimal cut-points were estimated by maximizing the discriminative ability of the logistic model with the dependent variable being the occurrence of disease control and the Cox model for PFS and OS.

Main effects and second-order interactions of each marker with sex were included in a multiple logistic regression model for the response outcome and in a multiple Cox proportional hazards regression model for the survival outcome. No deviation from the proportional hazard assumption was found by the Grambsch and Therneau statistical test.

Backward elimination using the Bayesian Information Criterion (BIC) was applied for selecting all variables independently associated with the outcome. The results were displayed in terms of odds ratios and hazard ratios together with 95% CI.

All statistical tests used a two-sided 5% significance level. Statistical analyses were performed using RStudio (RStudio: Integrated Development for R. RStudio, Inc., Boston, MA).

## Results

Between April 2020 and July 2022, 169 patients were screened at four Italian cancer centers: four patients were screening failure, four not evaluable for treatment response analysis due to early death, and 161 (92 MM and 69 NSCLC patients) were evaluable for the ORR. The patient population for survival analysis consisted of 165 patients.

Patient baseline characteristics were representative of an unselected population of MM and NSCLC patients, not previously treated with ICIs (Table 1 and Additional file 2: Tables S2 and S3). At the data cut-off (August 1, 2023) the median follow-up was 23 months (IQR 17.4–27.8). Males accounted for 60.9% of the treated population. The median age was 71 years, 94.3% ECOG PS 0–1, 88.7% stage IV disease, without differences in the two sexes. Seventy-nine (49.1%) patients received nivolumab while the remaining 58 patients received pembrolizumab (32.3%) or atezolizumab (3.7%); twenty-four (14.9%) NSCLC patients received chemoimmunotherapy. The distribution between males and females among the treatment groups was equally represented (Table 1).

**Table 1:** Patient characteristics for the overall study population

All patients		Male (N = 98)	Female (N = 63)	Total (N = 161)	p value
Age	Median (Q1, Q3)	72.0 (63.2, 77.0)	67.0 (60.0, 74.5)	71.0 (62.0, 77.0)	*0.1590*
Diagnosis	NSCLC	45 (45.9%)	24 (38.1%)	69 (42.9%)	*0.3280*
	Melanoma	53 (54.1%)	39 (61.9%)	92 (57.1%)	
ECOG Performance Status	N-Miss	0	1	1	
	0	52 (53.1%)	33 (53.2%)	85 (53.1%)	*0.9400*
	1	40 (40.8%)	26 (41.9%)	66 (41.2%)	
	2	6 (6.1%)	3 (4.8%)	9 (5.6%)	
Prior systemic treatment	N-Miss	0	1	1	
	No	84 (85.7%)	51 (82.3%)	135 (84.4%)	*0.5570*
	Yes	14 (14.3%)	11 (17.7%)	25 (15.6%)	
Current stage	N-Miss	2	0	2	
	III	11 (11.5%)	7 (11.1%)	18 (11.3%)	*0.9460*
	IV	85 (88.5%)	56 (88.9%)	141 (88.7%)	
Treatment	Nivolumab	46 (46.9%)	33 (52.4%)	79 (49.1%)	*0.4670*
	Pembrolizumab	30 (30.6%)	22 (34.9%)	52 (32.3%)	
	Atezolizumab	4 (4.1%)	2 (3.2%)	6 (3.7%)	
	Chemo-immuno	18 (18.4%)	6 (9.5%)	24 (14.9%)	
RECIST	CR	8 (8.2%)	4 (6.3%)	12 (7.5%)	*0.3010*
	PR	34 (34.7%)	14 (22.2%)	48 (29.8%)	
	SD	21 (21.4%)	19 (30.2%)	40 (24.8%)	
	PD	35 (35.7%)	26 (41.3%)	61 (37.9%)	
Progression of disease	No	33 (33.7%)	18 (28.6%)	51 (31.7%)	*0.4970*
	Yes	65 (66.3%)	45 (71.4%)	110 (68.3%)	
Progression free survival (months)	Median (95%CI)	7.6 (6.1,12.5)	8.1 (3.3,10.6)	8.1 (6.1,9.8)	*0.9826*
Status	Alive	41 (41.8%)	28 (44.4%)	69 (42.9%)	*0.7440*
	Death	57 (58.2%)	35 (55.6%)	92 (57.1%)	
Overall survival (months)	Median (95%CI)	13.9 (9.9,23.5)	18.9 (9.5,22.6)	16.7 (11.4,21.6)	*0.6118*
Follow-up	Median (Q1, Q3)	23.1 (17.4,29.4)	21.8 (16.8,27.6)	23.0 (17.4,27.8)	

### Activity and efficacy results in the two cohorts of patients

The ORR was 37.3% in the whole cohort, with 12 complete responses (7.5%), achieved only by MM patients. ORR was 43.4% and 29.0% in MM and NSCLC patients, respectively. Moreover, 62.1% of the population obtained DCR with a similar percentage in the two cohorts: 61.9% for MM and 62.3% for NSCLC patients (Table 1, Additional file 2: Tables S2–S3). The ORR reached in males was better than in females (42.9% vs 28.5%, respectively), however without any statistical significance (p = 0.067), as well for DCR (p = 0.478) (Table 1).

At the last data cut-off (August 1, 2023), 69 (42.9%) patients were still alive, 51 (31.7%) of them with no evidence of progression (Table 1). No significant difference in terms of PFS and OS was shown between males and females and between the two cohorts. Overall, the median PFS was 8.1 months (95% CI 6.1, 9.8), 7.6 months (95% CI 6.1, 12.5) for males and 8.1 (95% CI 3.3, 10.6) for females. The median OS was 16.7 months (95% CI 11.4, 21.6), 13.9 months (95% CI 9.9, 23.5) for males and 18.9 (95% CI 9.5, 22.6) for females (Table 1 and Additional file 2: Figure S1). In the MM cohort, the median PFS was 9.8 months (95% CI 6.9, 17.1), 13.4 months (95% CI 6.9, 26.7) for males and 9.6 (95% CI 3.0, not reached (NR)) for females (Additional file 2: Table S2 and Additional file 2: Figure S2A); the median OS was 24.2 months (95% CI 18.4, NR) with no differences between males (24.2 months, 95% CI 13.4, NR) and females (22.6 months, 95% CI 11.8, NR) (Additional file 2: Table S2 and Additional file 2: Figure S3A). In the NSCLC cohort, the median PFS was 6.3 months (95% CI 3.8, 8.8), 6.1 months (95% CI: 3.1, 8.8) for males and 7.1 months (95% CI: 2.9, 9.8) for females (Additional file 2: Table S3 and Additional file 2: Figure S2B) while the median OS was 9.7 months (95% CI 6.6, 14.0), 9.7 months (95% CI 4.5, 13.9) for males and 13.8 months (95% CI 5.6, 17.6) for females (Additional file 2: Table S3 and Additional file 2: Figure S3B).

### Sex-specific biomarker distribution according to response

The exploration of potential variation in baseline cytokine levels between males and females in the whole population revealed no sex-related differences (Additional file 2: Table S4), while we observed statistically significant lower values of IL-6 (p < 0.001), IL-8 (p = 0.019) and sPD-L1 (p = 0.006) in the MM cohort compared with the NSCLC cohort (Additional file 2: Table S5).

**Fig. 2:**
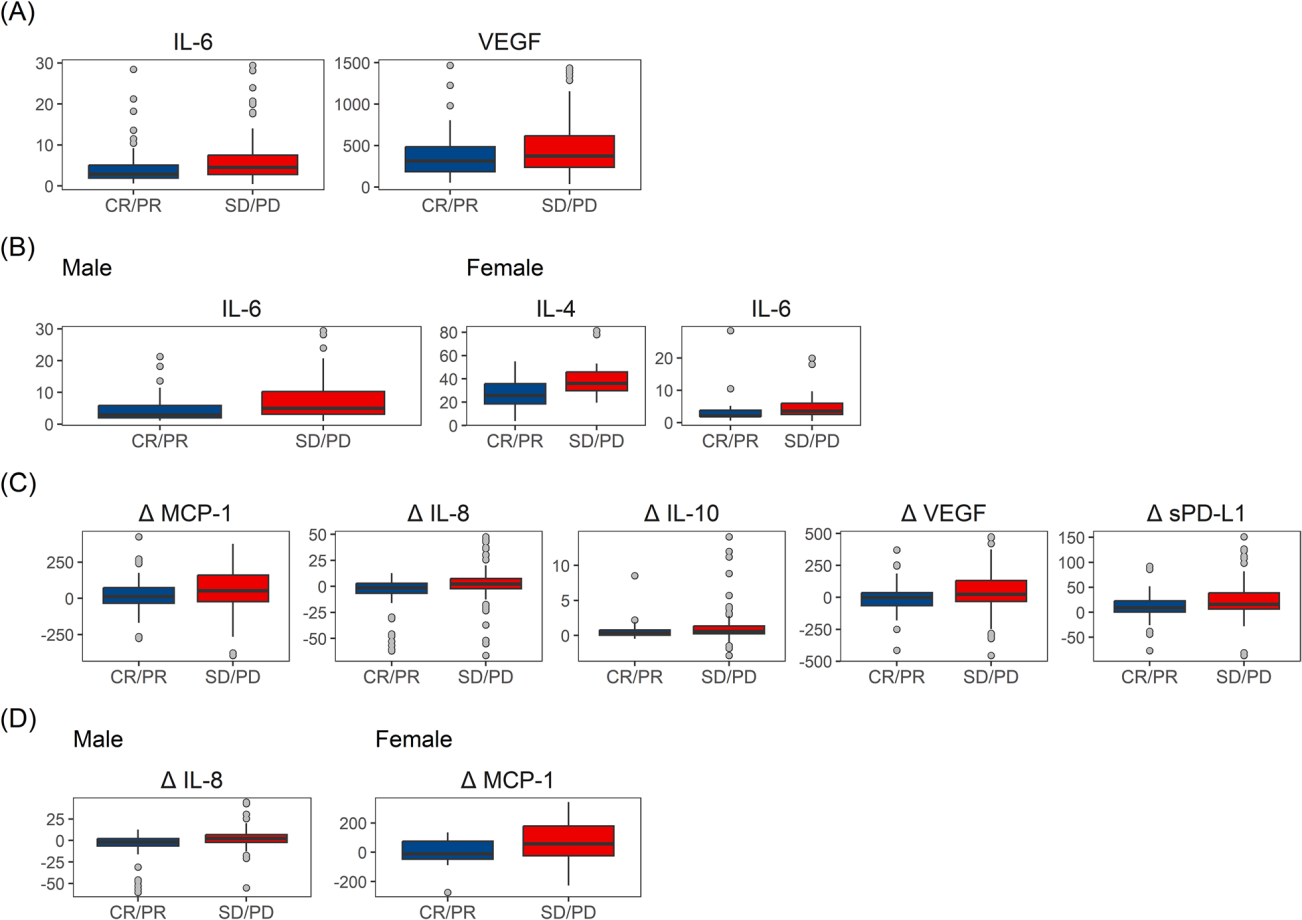
Boxplot of circulating levels of biomarkers (pg/ml) in patients achieving objective response (CR/PR) according to RECIST 1.1 criteria. Baseline values for the total population (**A**) and according to sex (**B**). Changes at cycle 2 from baseline values for the total population (**C**) and according to sex (**D**)

At baseline, IL-6 and VEGF were significantly lower in responders with respect to SD/PD patients (IL-6 median (IQR): 2.9 (1.9, 5.6) vs 4.9 (2.8, 8.6), p = 0.002, and VEGF median (IQR): 318.0 (188.5, 496.8) vs 396.0 (245.0, 663.0), p = 0.028) (Fig. 2A), with no differences according to sex. Interestingly, IL-4 was significantly underexpressed only in female responder patients (median IQR: 25.9 (18.6, 35.9) vs 36.7 (30.3, 47.9), p = 0.0070) (Fig. 2B). The disease control rate was associated, at baseline, with lower serum levels of IL-6, IL-8, and IL-10, with no differences between sexes (Additional file 2: Figure S4A). VEGF was significantly lower in male patients with DCR (median (IQR): 314.0 (186.0, 486.5) vs 387.0 (254.0, 970.0), p = 0.0300), while females achieving DCR had a lower expression of IL-4 (median (IQR): 28.6 (22.6, 37.9) vs 37.3 (33.8, 50.0), p = 0.0020 (Additional file 2: Figure S4B).

Observing the changes from baseline to cycle 2, we found a statistically significant reduction in IL-10 (median (IQR): 0.3 (0.0, 0.8) vs 0.6 (0.2, 1.3), p = 0.0370), VEGF (median (IQR): − 2.0 (− 71.0, 36.5) vs 41.5 (− 27.5, 167.5), p = 0.0050), and sPD-L1 (median (IQR): 9.1 (0.3, 22.8) vs 15.9 (6.2, 38.7), p = 0.0320) in responders compared to not responder patients, regardless of sex (Fig. 2C). Of note, males with ORR show a significant reduction in IL-8 levels (median (IQR): − 1.7 (− 6.8, 2.2) vs 2.7 (− 1.5, 9.3), p = 0.0001), whereas a statistically significant reduction was observed in MCP-1 (median (IQR): − 3.5 (− 43.5, 89.2) vs 104.0 (13.5, 193.0), p = 0.0420) in responder females (Fig. 2D). By analyzing the changes in serum cytokine from baseline to cycle 2 in the whole population achieving DCR, lower levels of IL-8 and IL-10 were observed compared to PD patients. Furthermore, male patients showed a significant decrease in sPD-L1, while female patients showed a significant decrease in IL-1β and VEGF (Additional file 2: Figure S4C-D).

### Sex-specific biomarkers predictive of activity (ORR and DCR)

Each cytokine was also analyzed for its association with treatment response as a categorical variable based on high and low cut-off levels. The optimal cut-off points for each marker were estimated by maximizing the discriminative capacity of the logistic model using the "minimax" criterion.

**Fig. 3:**
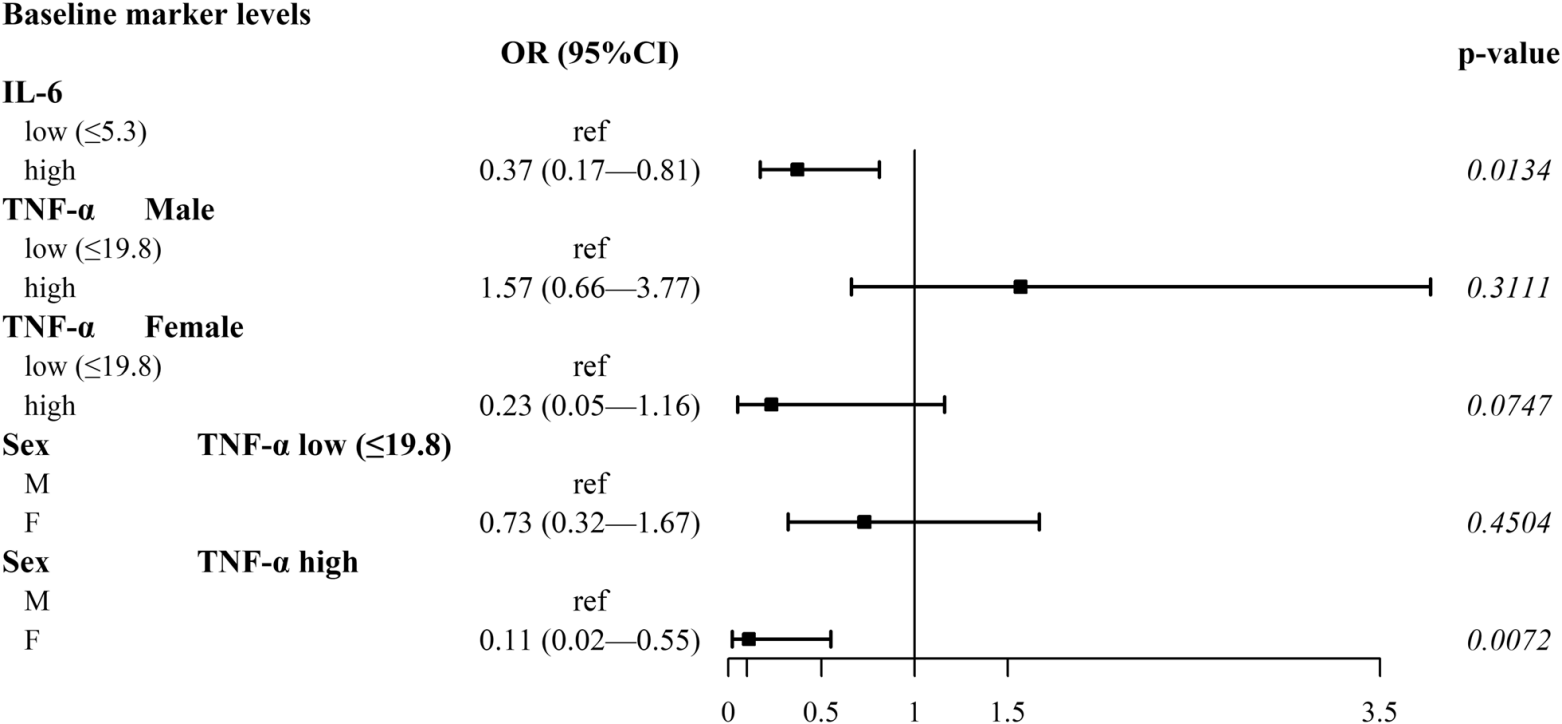
Forest plot of multiple logistic regression predicting ORR – baseline biomarker levels (pg/ml). An OR less than 1 indicates that the objective response is less likely to occur in the considered group compared to the "ref" group. OR: odds ratio; CI: confidence interval

In the multiple logistic analysis of baseline data, while IL-6 (cut-off point = 5.29 pg/ml) was associated with ORR regardless of sex, TNF-α (cut-off point = 19.75 pg/ml) was differently associated with ORR in males and females (p[interaction] = 0.0386) (Fig. 3). The probability of ORR was lower in females than in males (OR = 0.11, 95% CI 0.02, 0.55, p = 0.0072) in the presence of high TNF-α values (Fig. 3, Additional file 2: Table S6A). There were no significant interactions with sex for the ORR considering changes from baseline to cycle 2. However, increases in IL-8 (cut-off point = 3.23 pg/ml) and VEGF (cut-off point = 45.0 pg/ml) were associated with a lower probability of ORR (OR = 0.36, 95% CI 0.17, 0.77, p = 0.0086, and OR = 0.32, 95% CI: 0.15, 0.69, p = 0.0040, respectively) (Additional file 2: Table S6B). As far as DCR is concerned, high baseline values of IL-6 (cut-off point = 3.04 pg/ml) and IL-8 (cut-off point = 19.86 pg/ml) remained independently associated with a lower probability of DCR (Additional file 2: Table S7A), and the increase in sPD-L1 levels (cut-off point = 6.4 pg/ml) was significantly associated with a lower probability of response (OR = 0.40, 95%CI: 0.19, 0.85, p = 0.018) regardless of sex (Additional file 2: Table S7B).

### Sex-specific biomarkers predictive of efficacy (PFS and OS)

Each marker was then analyzed for association with PFS and OS as a categorical variable based on high and low levels. The optimal cut-off points for each marker were estimated by maximizing the discriminative power of the Cox model.

**Fig. 4:**
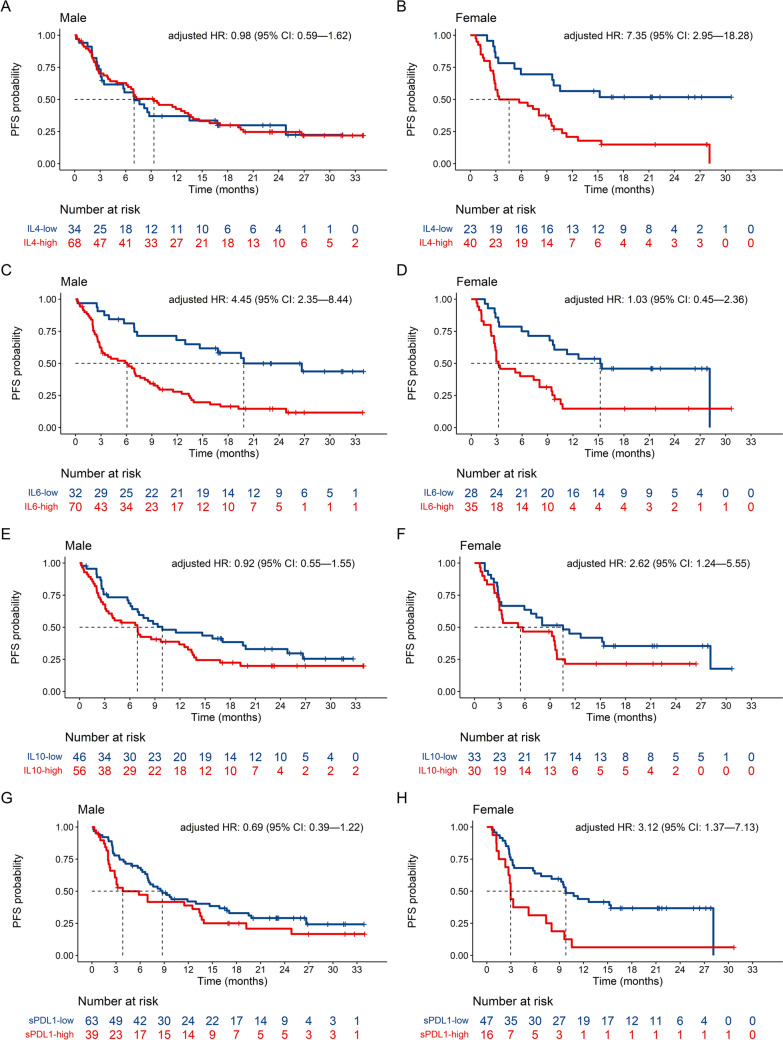
Kaplan–Meier curves for progression free survival (PFS) of baseline biomarker levels (pg/ml) interacting with sex in the multiple Cox regression model. HR: hazard ratio; CI: confidence interval

In the multiple Cox model of baseline data predicting PFS, elevated MCP-1 (cut-off point = 365 pg/ml, HR = 0.43, 95% CI: 0.28, 0.66) and IL-5 (cut-off point = 1.07 pg/ml, HR = 0.39, 95% CI: 0.25, 0.60) remained significantly associated with a longer PFS, while elevated IL-8 (cut-off point = 34.1 pg/ml, HR = 2.93, 95% CI: 1.77, 4.84) was significantly associated with a shorter PFS (Additional file 2: Table S8A). In relation to sex, poorer PFS were predicted by high levels of IL-4 (cut-off point = 28.6 pg/ml), IL-10 (cut-off point = 1.43 pg/ml) and sPD-L1 (cut-off point = 111 pg/ml) in females and by high levels of IL-6 (cut-off point = 2.97 pg/ml) in males (Fig. 4, Additional file 2: Table S8A).

**Fig. 5:**
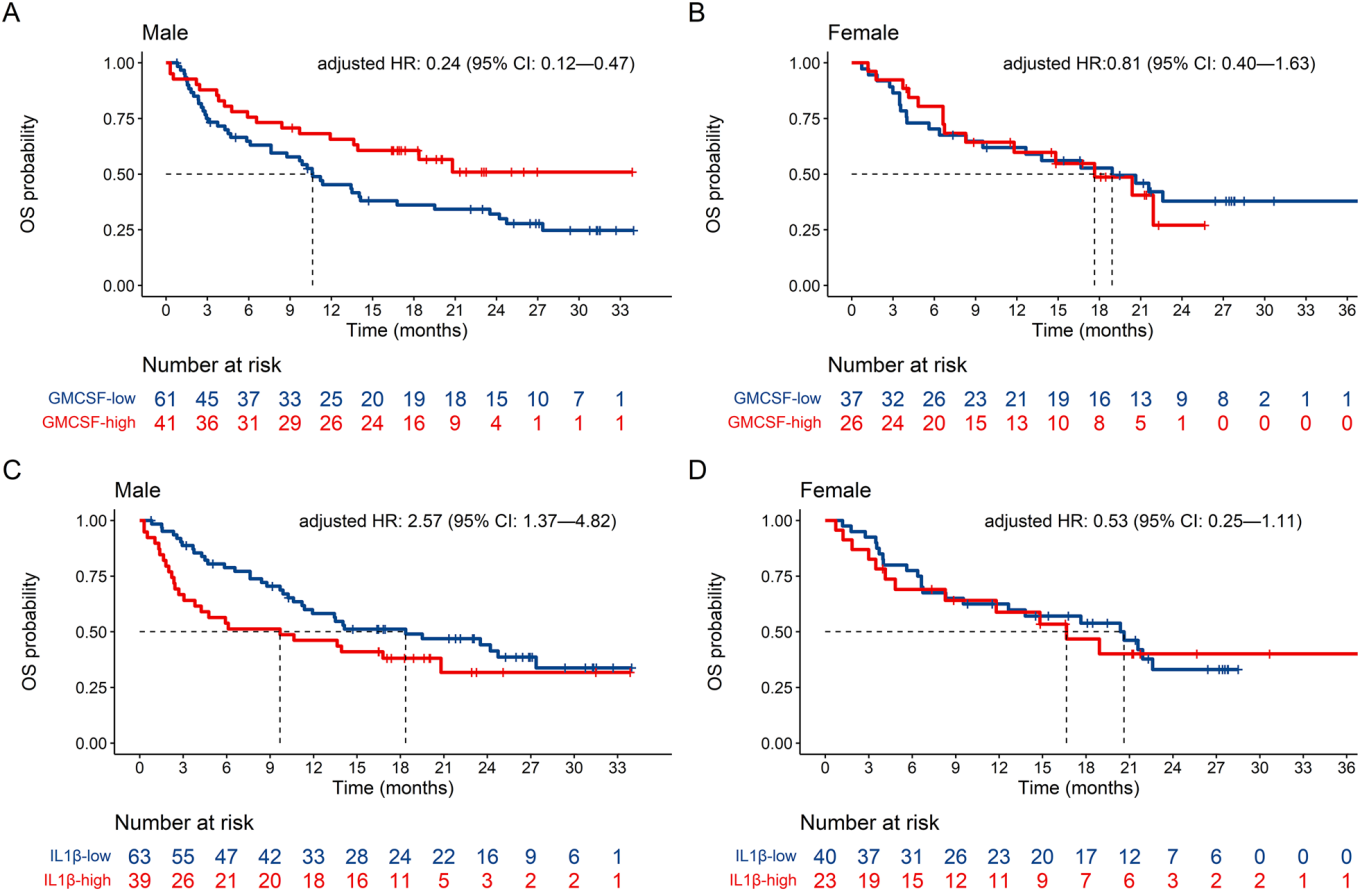
Kaplan–Meier curves for overall survival (OS) of baseline biomarker levels (pg/ml) interacting with sex in the multiple Cox regression model. HR: hazard ratio; CI: confidence interval

Elevated baseline MCP-1 (cut-off point = 365 pg/ml, HR = 0.52, 95% CI: 0.34, 0.82) was significantly associated with a longer OS, while elevated IL-6 (cut-off point = 2.9 pg/ml, HR = 4.35, 95% CI: 2.55, 7.41) and IL-8 (cut-off point = 34.1 pg/ml, HR = 3.13, 95% CI: 1.99, 4.94) were significantly associated with a worse OS (Additional file 2: Table S9A). Noteworthy in male patients, high levels of GM-CSF (cut-off point = 4.03 pg/ml) predicted a better OS while high levels of IL-1β (cut-off point = 1.22 pg/ml) predicted a worse OS in comparison with females (Fig. 5, Additional file 2: Table S9A).

Finally, considering the changes from baseline to cycle 2, there were no significant interactions with sex for both, PFS and OS. However, increases in IL-8 (cut-off point = 4.63 pg/ml) and VEGF (cut-off point = 69.0 pg/ml) were associated with a poorer PFS and the increase in TNF-α (cut-off point = 2.92 pg/ml) with a better PFS (Additional file 2: Table S8B). In parallel, increases in IL-4 (cut-off point = 0.88 pg/ml), IL-8 (cut-off point = 4.63 pg/ml) and VEGF (cut-off point = 69.0 pg/ml) were associated with a worse OS and the increase in IL-5 (cut-off point = 0.06 pg/ml) with a better OS (Additional file 2: Table S9B).

## Discussion

ICIs currently represent the standard of care for treating metastatic MM and non-oncogene-addicted NSCLC. During their clinical development, physicians have encountered several critical issues leading to the need for predictive factors for a better patient selection. In particular, the dissociation between response, PFS and OS is not an uncommon event, as is the occurrence of pseudoprogression, which should be distinguished from a real progression, and a fibrotic tissue from a persistence of disease. The identification of those patients who benefit most or are at higher risk of PD and early death, through a non-invasive method, remains an unmet medical need for several solid tumors [31]. In Italy, NSCLC and MM patient selection for ICIs (as single agent and/or in combination) is currently subjected to the assessment of PD-L1 expression on tumor cells, according to the pivotal trials design and to the regulatory authority indications. However, PD-L1 expression is considered an imperfect, dynamic and heterogeneous predictor, often unrelated to response [18, 19]. The serial analysis of multiple immune cytokines and of possible sex-related association could optimize the risk/benefit ratio of the treatment leading to a patient-centered approach in men and women. Moreover, the detection and monitoring of circulating biomarkers during therapy captures the dynamic and plastic relationship between disease and host immunity and reveals the immunological mechanisms of resistance and sensitivity, thus paving the way for more effective and fine-tuned treatments. This may overcome the critical issues encountered during the immuno-phenotyping and molecular analysis of the tumor samples and the dissection of the tumor microenvironment, not available for all metastatic sites, not easily repeatable and not always representative of the whole tumor-host relationship.

Available literature data about the differential efficacy and activity of ICIs in female compared with male patients are heterogeneous, although several studies and meta-analyses report a greater benefit in males, especially in the clinical context of MM [32–36]. In our series, we observed that ORR was higher in males than in females for both tumor types, while DCR and PFS were quite similar, and OS was better in females for both cancer types. Although not statistically significant, the differences observed in the ORR and OS could be considered clinically relevant and worthy of further investigation.

To our knowledge, this is the first translational prospective study evaluating a panel of 13 serum immune-cytokines in a real-world population including two different cancer types treated with ICIs, with the aim of identifying predictive markers of activity and efficacy, and their sex interaction.

No differences were observed in the baseline levels of circulating cytokines between the two sexes. A differential distribution of IL-4 levels in females achieving objective response and disease control, and of VEGF in males achieving disease control, was observed. Overall, higher levels of specific cytokines were suggestive of a worse antitumor immunity in a differential way between the two sexes.

Beyond the different distribution of IL-4 in female patients, where ICIs were found to be active, higher baseline levels were also correlated with shorter PFS in women, thus confirming a sex-related interaction for this biomarker.

IL-4 is involved in multiple immunological functions, both pro-tumor and anti-tumor depending on the context. In melanoma, it prompts a more unfavorable clinical course and the induction of immunological escape [37]. Indeed, the effect of the monoclonal inhibitor dupilumab in resolving nivolumab-induced bullous pemphigus has been reported [38], and its potential role in combination with anti-PD-1 in patients with high serum IL-4 levels may be worth investigating.

When we looked for a predictive correlation with ORR, only baseline levels of TNF-α, a cancer-promoting and immunosuppressive cytokine, reached statistical significance in responder females, confirming a worse antitumor immunity in presence of high baseline TNF-α levels as already described [39]. Although TNF-α antagonists (infliximab, etanercept, adalimumab and certolizumab) have also been used as rescue therapy for ICI-induced colitis, arthritis and pneumonitis, controversial preclinical and clinical data about the effect of TNF-α antagonists on patients survival are currently available [40, 41].

Furthermore, TNF-α has been reported to determine the differentiation of T cells in Th2 type through an increase of soluble IL-6 receptor (sIL-6r) and with sex-interactions [42]. IL-6 contributes to tumor promotion by the expansion and survival of malignant cells, neo‐angiogenesis, and inflammation, and it promotes expression of the T helper Th2 associated with IL-4. Differently from our previous report showing that higher baseline IL-6 levels in MM patients treated with anti CTLA-4 antibodies were independently related to a worse survival rate in females [43], in this case series a sex-related interaction of IL-6 levels and activity or efficacy of ICIs was not found. However, we found that baseline IL-6 were significantly higher in NSCLC than in MM patients and in men than in women and were significantly associated with a lower ORR, DCR, PFS and OS in the overall population, with a significantly worse PFS in males. Our data seem in agreement with available literature [44–46] suggesting that higher baseline IL-6 levels, and their increase during ICI treatment, are predictive of a worse outcome. The use of IL-6 or IL-6r inhibitors, such as sarilumab or tocilizumab, is approved for the treatment of immune related toxicities, autoimmune diseases such as arthritis rheumatoid, and of cytokine release syndrome from CAR-T or Tebentafusp. A synergistic effect combining IL-6 inhibitors and ICI has been reported [47–49] and deserves to be further developed.

Moreover, through the induction of STAT3, IL-6 also induces the expression of angiogenic molecules, including VEGF [50]. The interconnection between neo-angiogenesis and an immunosuppressive microenvironment is already recognized [51], and the combination of antiangiogenics with ICIs has been explored with heterogeneous results in different solid tumors [52, 53]. Indeed in melanoma patients, the association of anti-PD-1 with anti-VEGF has proved futile (LEAP-003 trial) [54], suggesting that tumor selection and patient stratification, according to the immunological targets, is crucial. We observed that, during ICI therapy, an early decrease in serum VEGF levels increased the probability of ORR and DCR in both sexes, with a significantly better DCR in women; conversely, an increase translated into a poorer PFS and OS.

We also found that baseline IL-8 and sPD-L1 were significantly higher in NSCLC than in MM patients and, at multiple Cox regression, IL-6 and IL-8 were independently associated with PFS and OS, while sPD-L1 was associated with PFS. In particular, we observed an independent shorter PFS in women with sPD-L1 baseline levels > 111 pg/ml and the early decrease was significantly associated with an improved, sex-independent, ORR and DCR. sPD-L1 is produced and released by neoplastic cells and high baseline levels were observed in patients with lower ORR, PFS and OS [55–58]. The expression of sPD-L1 in advanced NSCLC patients was significantly upregulated compared to the healthy control; it correlated significantly with abdominal organ metastasis and with a worse prognosis [59]. In metastatic melanoma, elevated baseline serum levels of PD-1 and/or PD-L1 are significantly correlated with a lower rate of best overall response (BOR), PFS and OS at multivariable Cox regression [60].

While there is no sex-related association between baseline IL-8 circulating levels and activity or efficacy of ICIs, a significant reduction in IL-8 and MCP-1 was observed at cycle 2 in responder males and females, respectively. A correlation between IL-8 serum levels and reduced clinical benefit of ICIs in patients with melanoma, NSCLC, hepatocellular carcinoma and renal cancer was first reported by Sanmamed et al*.*, suggesting its use as a prognostic biomarker in monitoring anticancer therapy [61]. High IL-8 serum levels mirror an unfavorable tumor microenvironment characterized by the infiltration of myeloid suppressor cells, neutrophils, monocyte, pro-angiogenic molecules, and an impaired T cell cytotoxicity and a recent meta-analysis concluded that IL-8 could be a therapeutic target, not only in melanoma and NSCLC, but also in other tumors [62, 63]. Many agents targeting IL-8 and IL-8 receptors, and many combined strategies are under clinical investigation: monoclonal antibodies, neutralizing antibodies, direct antagonists, antibodies anti-CXCR1 and -CXCR2, in different tumor types, with the aim of increasing the anti-tumoral efficacy [64, 65].

Decreases in IL-8 and MCP-1 were related to a better outcome in NSCLC patients receiving anti PD-1 therapy [66, 67] underlining the fact that course-tracing is more precise than baseline evaluation.

MCP-1 binding to its receptor CCR2 activates monocytes and induces leukocyte infiltration, as well as T-cell proliferation; it acts also as a regulator in the polarization of Th0 cells toward a Th2 phenotype and, coupled with IL-4 signaling, it could contribute to tumor growth and metastasis [68, 69]. The majority of studies reported MCP-1 related to tumor progression, clinical aggressiveness, and the promotion of metastasis, with a prevalent worse effect for digestive tract, urogenital, and head neck tumors, but no detrimental effect has been observed in pulmonary tumors [70], and a reduced metastatic potential has been reported in a murine model of colon cancer and in pancreatic cancer [71, 72]. As reported above, MCP-1 interacting with IL-4 could lead to an immune suppressive effect and we observed that higher baseline levels of IL-4 impair the probability of response, with a significantly worse effect in women.

Overall, these results could be used to set up a panel of the most significant cytokines (IL-8, IL-6, sPD-L1, VEGF, IL-4, TNF-α) to be tested prospectively, balanced by clinical risk factors and in different tumors, with the aim of selecting the best treatment choice according to the patient-tumor relationship. For clinical purposes, an easy, sensitive, and reproducible method for cytokine monitoring is desirable. As ELLA utization can further potentially reduce the number of steps and the time required to perform the biomarker analysis, an exploratory validation study investigating the agreement between Luminex and ELLA technologies in the measurement of relevant cytokines is currently ongoing.

Our findings may also be used to answer other undefined issues, such as optimal treatment duration; indeed, the circulating values of specific cytokines may allow us to descale the therapy in patients with more favorable profiles and to increase it in those whose profiles are less favorable.

The significance observed by analyzing two cancers suggests that our data can be tested and extended to other neoplasms. Moreover, the cytokines of major interest were not subjected to baseline differences between melanoma and NSCLC, and the role of some of them were consistent with previous studies [73].

Nonetheless, further validation using larger sample sizes and across multiple cancer types is necessary to consider the identified cytokines as pan-tumor predictive biomarkers for immunotherapy.

Our study included patients treated according to clinical practice, therefore a potential limitation of our observations is that no data are reported in patients receiving the dual checkpoint inhibition (anti-PD-1 plus anti-CTLA-4); indeed during almost the whole enrolling period the combination was not reimbursed in our Country. Although this treatment is now available, it is restricted to a small selected patient subgroup, making our data still representative of the current clinical context.

On the other hand, the main strength of the present study is that it was conducted in a real-word setting, producing results with external validity.

Future perspectives include the evaluation of the cytokine levels and their fluctuation in relation to the toxicity, and the potential interaction with different drugs (such as steroids, immunosuppressive agents, antibiotics, antiviral and anti-inflammatory agents), infections, comorbidities and metastatic sites.

## Conclusions

This is the first translational prospective study investigating the predictive and prognostic values of a large panel of circulating cytokines and their sex-interaction in MM and NSCLC patients receiving ICIs, at the baseline and at different timepoints. We identified selected cytokines differentially expressed before treatment start as well as their changes at the second cycle of treatment in responder males and females, thus suggesting a potential role as biomarkers to be prospectively validated as selection criteria in upcoming clinical trials and across different cancer types.

### Supplementary Information

**Additional file 1: **Supplementary methods.

**Additional file 2: Figure S1.** Kaplan-Meier curves for progression free survival (PFS) (**A**) and overall survival (OS) (**B**) of total population and stratified by sex. **Figure S2**. Kaplan-Meier curves for progression free survival (PFS) of Melanoma patients (**A**) and NSCLC patients (**B**) of all population and stratified by sex. **Figure S3**. Kaplan-Meier curves for overall survival (OS) of Melanoma patients (**A**) and NSCLC patients (**B**) of all population and stratified by sex. **Figure S4**. Boxplot of circulating levels of biomarkers (pg/ml) in patients achieving disease control (CR/PR/SD) according to RECIST 1.1 criteria. Baseline values for the total population (**A**) and according to sex (**B**). Changes at cycle 2 from baseline values for the total population (**C**) and according to sex (D). **Table S1.** Italian Medicines Agency indications for Immune Checkpoint Inhibitors in Melanoma and NSCLC patients, at the time of study enrollment. **Table S2**. Patient characteristics for the melanoma study population. **Table S3.** Patient characteristics for the NSCLC study population. **Table S4**. Baseline levels of biomarkers (pg/ml) according to sex. **Table S5**. Baseline levels of biomarkers (pg/ml) according to diagnosis. **Table S6. **Multiple logistic regression predicting the objective response rate for biomarker baseline levels (pg/ml) (**A**), and variation between cycle 2 and baseline (**B**). **Table S7**. Multiple logistic regression predicting disease control rate of biomarker baseline levels (pg/ml) (**A**), and variation between cycle 2 and baseline (**B**). **Table S8**. Multiple Cox regression predicting the progression free survival (PFS) for biomarker baseline levels (pg/ml) (**A**), and variation between cycle 2 and baseline (**B**). **Table S9**. Multiple Cox regression predicting the overall survival (OS) for biomarker baseline levels (pg/ml) (**A**), and variation between cycle 2 and baseline (**B**).

## Data Availability

The datasets for this study are available at 10.5281/zenodo.10219235.

## Abbreviations

AIFA: Agenzia Italiana del Farmaco (Italian Medicines Agency)
AJCC: American Joint Committee on Cancer
CD4: Clusters of differentiation 4
CI: Confidence interval
CR: Complete response
CTLA-4: Cytotoxic T-lymphocyte antigen 4
DCR: Disease control rate
ECOG-PS: Eastern cooperative oncology group performance status
EGF: Epidermal growth factor
FGF: Fibroblast growth factor
G-CSF: Granulocyte-colony stimulating factor
GM-CSF: Granulocyte macrophage colony stimulating factor
GRO: Growth-regulated protein
HR: Hazard ratio
ICI: Immune checkpoint inhibitor
IFN: Interferon
IL: Interleukin
IL1-ra: Interleukin-1 receptor antagonist
IP-10: Interferon-inducible protein-10
IQR: Interquartile range
MCP-1: Monocyte chemotactic protein-1
MHC: Major histocompatibility complex
MIP: Macrophage inflammatory protein
MM: Malignant melanoma
NR: Not reached
NSCLC: Non-small cell lung cancer
ORR: Objective response rate
OS: Overall survival
PFS: Progression free survival
PD: Progression disease
PD-1: Programmed cell death protein 1
PD-L1: Programmed cell death ligand 1
PR: Partial response
SD: Stable disease
sPD-L1: Soluble programmed death ligand 1
TNF: Tumor necrosis factor
VEGF: Vascular endothelial growth factor
