# Supplementary material for: Sex-related differences in serum biomarker levels predict the activity and efficacy of immune checkpoint inhibitors in advanced melanoma and non-small cell lung cancer patients
Source: J Transl Med. 2024 Mar 5;22:242. doi: 10.1186/s12967-024-04920-6 (PMC10916307; doi:10.1186/s12967-024-04920-6)
Supplement: Supplementary file 1 — Additional file 1: Supplementary methods. [file 12967_2024_4920_MOESM1_ESM.docx]

**SUPPLEMENTARY MATERIAL**

**METHODS**

***Additional details about blood sample collection and processing***

Taking in consideration that pre-analytical variations in cytokine stability can occur in blood-derived matrix samples because of the variability in whole-blood processing procedures, standard operating procedures (SOPs) were used for the blood sample collection, processing and storage with the aim to generate robust and reproducible data. The procedure also includes the preparation of EDTA and CTAD plasma in order to establish a blood biobank. The set of samples were collected at baseline and in each therapy cycle prior to any receipt of ICI.

***Standard Operating Procedure for Blood Sample Collection***

*Material*

- A test tube rack to hold blood collection tubes which are drawn from each patient
- Sterile, disposable 21-gauge butterfly needles
- BD Vacutainer Safety-Lock set for blood collection
- Disposable plastic gloves
- Sterile alcohol swabs
- A tourniquet
- Bandages ("Band Aids")
- Blood collection tubes

- CTAD plasma tube:
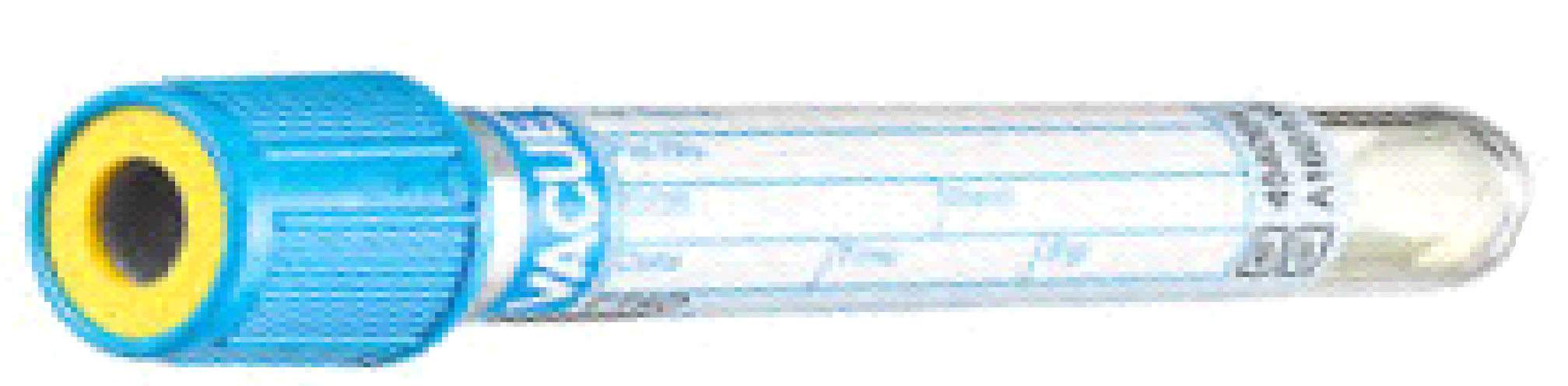

pre-chilled VACUETTE® TUBE, blue stopper, containing CTAD (sodium citrate, theophylline, adenosine and dipyridamole) 0.11M concentration for plasma; volume 3.5 ml; dimensions 13x75mm. Greiner Bio-One; COD. 454064. CTAD tubes should be stored at room temperature and protected from light.

- SST Serum tube:
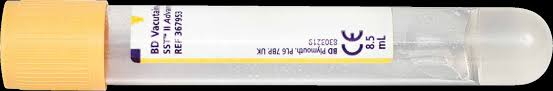


SST II serum tube, yellow stopper, containing SST™ II advance, containing an activator of coagulation (micronized silica) and separating gel; volume 8.5 ml; dimensions 16x100mm. Becton Dickinson (BD); COD. 367953.

- K_2_EDTA plasma tube:
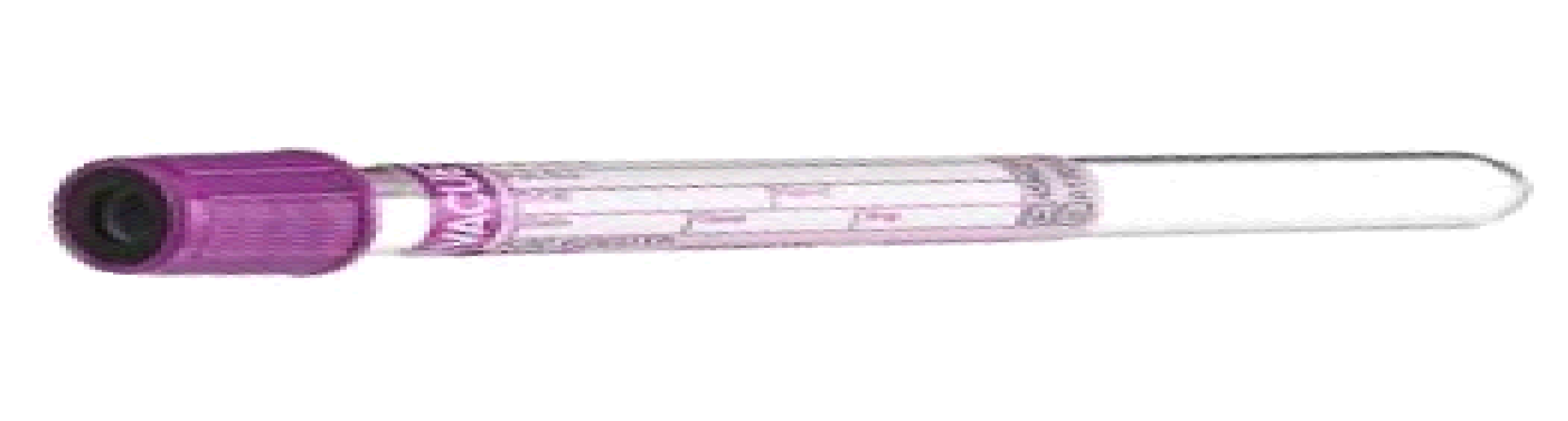


VACUETTE® TUBE PREMIUM lavender stopper, containing K_2_EDTA coating (spray 1.2-2 mg/ml of blood); volume 6 ml; dimensions 13x100mm. Greiner Bio-One; COD. 456023.

*Procedure*

1. Blood samples are collected by standard venipuncture techniques from all participants in the morning. Fasting is preferred but not required (a light breakfast is allowed). The venipuncture is performed with a 21-gauge butterfly needle with 12 inches of plastic tubing between the venipuncture site and the blood collection tubes.

1. All blood sample tubes are identified with a clearly marked label with study identification, unique patient identifier, date collection and specimen type (blood derived-matrix description). The labels are applied to the blood collection tubes for each patient just prior to blood collection.
2. About 21.5 ml of blood are drawn from each patient using vacutainer tubes, in the following sequence:
   - 3.5 ml of blood in each of the 2 blue top tubes (tot. 7 ml) for the preparation of CTAD plasma (that must be gently inverted 4-5 times immediately after filling).
   - 8.5 ml of blood in each of the yellow top tubes for the preparation of serum (that must be gently inverted 5-6 times immediately after filling).
   - 6 ml of blood in each of the lavender top tubes for the preparation of EDTA plasma (that must be gently inverted 8 -10 times immediately after filling).
3. The identifying information is checked on the form (Sample Log Sheet) and label to make sure that the specimen belongs to the patient identified on the labels.
4. The exact time of sample collection are recorded on the Sample Log Sheet.

***Standard Operating Procedures for Serum Processing***

Materials:

- Specimen Collection Kit
- Refrigerated centrifuge with brake off feature
- Tube racks
- Pipettes
- Cryovials
- 10x10 freezer boxes

1. Label vacutainer tubes and transfer vials with patient unique ID, date of collection, and specimen type.

2. Using a Vacutainer adapter and double ended needle, collect blood into one BD Vacutainer® Serum Separator Tubes (SST^TM^ II Advance) (Becton, Dickinson and Company, Franklin Lakes, NJ, USA; 8.5ml, yellow cap, Hemogard ^TM^ closure cap, PET, size 16x100mm, micronized silica as clot activator and gel separator).

3. The serum tube must sit upright in a rack at room temperature for 30 - 45 minutes for clot formation to occur and must be processed immediately at 4°C.

4. Ensure serum tubes remain cold at all times while processing (either on ice or in cold centrifuge). Make sure to aliquot quickly once serum is separated.

5. Place vacutainer tubes in centrifuge at 4 °C for 15 min at 2500 g (brake off), making sure to record time of processing in the Sample Log Form

6. Using a Pasteur pipette, place serum in a 15 ml conical tube.

7. Using a p1000 pipette, transfer 500 ul of serum into labelled aliquot tubes (labelled cryovials).

8. Close tubes tightly and store in 10x10 boxes at -80°C.

**Biomarker selection and measurement**

***Biomarker selection***

Our conceptual-methodological framework to select the biomarkers to be analysed as well as the more appropriate method to measure them consisted of two-steps:

1. the screening of different measurable molecules in serum and plasma by using different multi-marker assays;
2. a literature search to identify cytokines which may have potential involvement with melanoma development, immunotherapy efficacy and gender differences in oncological responses.
3. *Screening of different measurable molecules*

In an initial phase of the study, activities related to the screening of measurable molecules in serum and plasma were carried out. Different panels of markers were measured in the serum and CTAD plasma of the first group of patients enrolled at the study in order to detect which markers are potentially measurable in the two matrices and also to identify the blood matrix and the assay method that allows the adequate determination of a greater number of biomarkers. At this stage: (i) a screening of measurable molecules in two biological matrices, serum and CTAD plasma, was carried out, and the serum was selected as the matrix to be evaluated throughout the study; (ii) 3 commercial multiplex kits and one Simple-Plex cartridge, different regarding analytical sensitivity, panel of selected cytokines and manufacturing company were evaluated. In all the steps of this process, considering that cytokines can be classified in the category of "fragile markers" (as they are unstable and easily degraded), the state-of-art of research on cytokines analysis was taken into consideration.

Globally, 28 immune-related biomarkers (IL-1⍺, IL-1β, IL-1ra, IL-2, IL-4, IL-5, IL-6, IL-8, IL-10, IL-12(p70), IL-13, IL-17A, EGF, FGF-2, G-CSF, GM-CSF, GRO-⍺, IFN-⍺, IFN-γ, IP-10, MCP-1, MIP-1⍺, MIP-1β, soluble PD-L1, TNF-⍺, VEGF, Eotaxin, Fractalkine) were subjected to screening in 38 samples (analysed in duplicate) collected at baseline and during immunotherapy from 10 advanced melanoma or NSCLC patients, at different time points. Patient and sample characteristics are described elsewhere [Suppl. Ref. 1]. This analysis allowed the identification of 25 molecules measurable in at least 30% of serum samples from patients affected by melanoma and advanced NSCLC, including IL-1β, IL-1ra, IL-2, IL-4, IL-5, IL-6, IL-8, IL-10, IL-12(p70), IL-13, IL-17A, EGF, G-CSF, GRO-α factor, GM-CSF, IFN-α, IFN-γ, IP-10, MCP-1, MIP-1β, sPD-L1, TNF-α, VEGF, eotaxin, fractalkine.

1. *Literature search to identify cytokines of interest*

Next, a literature search was performed to identify cytokines which may have potential interest to the study, while involved with melanoma development, immunotherapy efficacy and gender-specific differences in immune response. Therefore, with the assessment of the scientific evidence [Suppl. Ref. 2-7], and with the evaluation of the availability of the molecules in the panels customizable and with adequate sensitivity, 13 markers were selected to be assayed in the samples of the entire series, including IL-1β, IL-2, IL-4, IL-5, IL-6, IL-8, IL-10, GM-CSF, TNF-⍺, MCP-1, IP-10, VEGF and sPD-L1.

Further details on method validation and screening activities are published elsewhere [Suppl. Ref. 1].

***Additional details about Biomarker Measurement***

For analysis using xMAP multiplexing Luminex technology, immediately prior to the initiation of study measurements the Bio-Plex platform underwent a complete on-site maintenance cycle and operational qualification by Bio-Rad technician. Daily calibration and weekly validation were performed by researchers during the entire analytical period. During the assays, 96 well-plates were loaded with antigen-specific capture antibody-coated magnetic beads. Next, samples, standards and blanks were added and incubated overnight together with the beads. Biotinylated detection antibodies were added as a second step. The antibody/antigen complex was then visualised by incubation with streptavidin-conjugated R-phycoerythrin. In between all steps of the protocol, a washing procedure was performed by means of a magnetic plate washer (Bio-Plex Pro Wash Station Magnetic Plate Washer; Bio-Rad Laboratories Inc. Hercules, CA, USA). In addition to the normal start-up procedures (i.e., a series of fluidic functions) suggested by the manufacturer, three additional wash cycles (using deionized water to clean tubing) and one priming cycle with sheath fluid were performed to prepare for bead analyses before processing experimental plates as suggested by Hulse et al. (2004) [Suppl. Ref. 8]. Read-out was performed with the Bio-plex 200 system (Bio-Rad Laboratories Hercules, California, USA; xMAP multiplexing technology, Luminex): the instrument was calibrated, a standard curve was created, and the observed concentrations of samples were calculated. Raw and background-subtracted signal levels are reported in relative fluorescence units (RFU). Concentrations of the proteins in pg/ml were determined using five parameter log curves generated by the Bio-Plex Manager 6.0 software.

Biomarker levels and relative inter-assay coefficient of variation (CV%) measured in a pool of patient samples (used as positive control) and expressed as the mean of values obtained in 20 different runs were: IL-1β: 2.74 pg/ml, CV% 15.73; IL-2: 3.77 pg/ml, CV% 17.85; IL-4: 42.03 pg/ml, CV% 15.38; IL-5: 0.95 pg/ml, CV% 13.71; IL-6: 5.56 pg/ml, CV% 10.3; IL-8: 40.32 pg/ml, CV% 6.64; IL-10: 3.01 pg/ml, CV% 12.52; GM-CSF: 6.6 pg/ml, CV% 19.6; TNF-⍺: 19.8 pg/ml, CV% 8.77.

For Simple Plex Assay Protocol on ELLA platform, diluted samples and wash buffer were added to appropriate wells on the ELLA cartridge. Sample results were reported using Simple Plex Runner 3.9.0.28 for Windows 10 (ProteinSimple). A barcode scanner was used to identify the cartridge (format, analytes, etc.) and automatically load lot-specific factory-calibrated standard curves that are embedded in each cartridge barcode. Sample codes and dilution factors were input using Simple Plex Runner software, which facilitates proper data organisation and execution of automated scripts that define assay protocol and fluorescent scan parameters without user intervention. In fact, all immunoassay operations (including prime system, flow samples and split them into channels, sample incubation, wash, rehydrate and flow secondary antibody, wash, rehydrate and flow streptavidin dye conjugate, incubate, wash, scan) are processed automatically. At the conclusion of the assay, triplicate results, one per Glass Nano Reactor (GNR), for every analyte in each sample are automatically displayed. Raw (background-subtracted) signal levels are reported in relative fluorescence units (RFU) for each individual GNR, and mean RFU signal values, standard deviation, and coefficient of variance (CV%) are provided for triplicate GNRs. RFU values are automatically back-fit to barcode-embedded standard curves, and back-fit concentrations are multiplied by user-defined dilution factors to provide calculated biomarker levels in pg/ml for each sample.

Biomarker levels and relative inter-assay CV% measured in a pool of patient samples (used as positive control) and expressed as the mean of values obtained in 20 different runs were: MCP-1: 506.67 pg/ml, CV% 2.49; IP-10: 272.56 pg/ml, CV% 2.5; VEGF: 383.33 pg/ml, CV% 6.09; sPD-L1: 106 pg/ml, CV% 2.91.

**Supplementary References**

1. Cappelletto E, Tiozzo Fasiolo L, Salizzato V, Piccin L, Fabozzi A, Contato A, Del Bianco P, Pasello G, Chiarion-Sileni V, Gion M, Fabricio ASC. Cytokine and soluble programmed death-ligand 1 levels in serum and plasma of cancer patients treated with immunotherapy: Preanalytical and analytical considerations. Int J Biol Makers. 2024, *in press.*
2. Blank CU, Haanen JB, Ribas A, Schumacher TN. CANCER IMMUNOLOGY. The "cancer immunogram". Science. 2016 May 6;352(6286):658-60. doi: 10.1126/science.aaf2834. (studied markers: VEGF, G-CSF, GM-CSF, IL-1 alpha, IL-1beta, IL-6, IL-17, Groalpha, PCR)
3. DeCotiis C, Hu Y, Greenberg AK, Huie M, Tsay JC, Pass H, Goldberg JD, Rom WN. Inflammatory cytokines and non-small cell lung cancer in a CT-scan screening cohort: Background review of the literature. Cancer Biomark. 2016;16(2):219-33. doi: 10.3233/CBM-150559. (studied markers: IP-10, IL-8, IL-6, TNF-alpha, IL-4, IL-5, IL-13, IL-17, VEGF)
4. Gabriele L, Buoncervello M, Ascione B, Bellenghi M, Matarrese P, Carè A. The gender perspective in cancer research and therapy: novel insights and on-going hypotheses. Ann Ist Super Sanita. 2016 Apr-Jun;52(2):213-22. doi: 10.4415/ANN_16_02_13. (studied markers: IL-1, IL-6, TNF-alpha, IL-10)
5. Klein SL, Flanagan KL. Sex differences in immune responses. Nat Rev Immunol. 2016 Oct;16(10):626-38. doi: 10.1038/nri.2016.90.(studied markers: IL-10, TNF, IFNgamma, IL-4, IL-10, IL17, CXCL8, CCL2, GM-CSF, IFN-alpha, IL-1beta, IL-4, IL-5, IL-10, TGF-beta, IL-2)
6. Mitsuhashi A, Okuma Y. Perspective on immune oncology with liquid biopsy, peripheral blood mononuclear cells, and microbiome with non-invasive biomarkers in cancer patients. Clin Transl Oncol. 2018 Jan 8. doi: 10.1007/s12094-017-1827-7. (studied markers: IL-10, IL-6, IL-8, TGF-beta, angiopoietin, sPD-L1)
7. Sharma G, Mauvais-Jarvis F, Prossnitz ER. Roles of G protein-coupled estrogen receptor GPER in metabolic regulation. J Steroid Biochem Mol Biol. 2018 Feb;176:31-37. doi: 10.1016/j.jsbmb.2017.02.012. (studied markers: TNF-alpha, MCP-1, IL-1beta)
8. Hulse RE, Kunkler PE, Fedynyshyn JP, Kraig RP. Optimization of multiplexed bead-based cytokine immunoassays for rat serum and brain tissue. J Neurosci Methods. 2004 Jun 15;136(1):87-98. doi: 10.1016/j.jneumeth.2003.12.023.
