# Supplementary material for: Sex-related differences in serum biomarker levels predict the activity and efficacy of immune checkpoint inhibitors in advanced melanoma and non-small cell lung cancer patients
Source: J Transl Med. 2024 Mar 5;22:242. doi: 10.1186/s12967-024-04920-6 (PMC10916307; doi:10.1186/s12967-024-04920-6)
Supplement: Supplementary file 2 — Additional file 2: Figure S1. Kaplan-Meier curves for progression free survival (PFS) (A) and overall survival (OS) (B) of total population and stratified by sex. Figure S2. Kaplan-Meier curves for progression free survival (PFS) of Melanoma patients (A) and NSCLC patients (B) of all population and stratified by sex. Figure S3. Kaplan-Meier curves for overall survival (OS) of Melanoma patients (A) and NSCLC patients (B) of all population and stratified by sex. Figure S4. Boxplot of circulating levels of biomarkers (pg/ml) in patients achieving disease control (CR/PR/SD) according to RECIST 1.1 criteria. Baseline values for the total population (A) and according to sex (B). Changes at cycle 2 from baseline values for the total population (C) and according to sex (D). Table S1. Italian Medicines Agency indications for Immune Checkpoint Inhibitors in Melanoma and NSCLC patients, at the time of study enrollment. Table S2. Patient characteristics for the melanoma study population. Table S3. Patient characteristics for the NSCLC study population. Table S4. Baseline levels of biomarkers (pg/ml) according to sex. Table S5. Baseline levels of biomarkers (pg/ml) according to diagnosis. Table S6. Multiple logistic regression predicting the objective response rate for biomarker baseline levels (pg/ml) (A), and variation between cycle 2 and baseline (B). Table S7. Multiple logistic regression predicting disease control rate of biomarker baseline levels (pg/ml) (A), and variation between cycle 2 and baseline (B). Table S8. Multiple Cox regression predicting the progression free survival (PFS) for biomarker baseline levels (pg/ml) (A), and variation between cycle 2 and baseline (B). Table S9. Multiple Cox regression predicting the overall survival (OS) for biomarker baseline levels (pg/ml) (A), and variation between cycle 2 and baseline (B). [file 12967_2024_4920_MOESM2_ESM.docx]

**SUPPLEMENTARY FIGURES AND TABLES**

***Supplementary Figure 1****. Kaplan-Meier curves for progression free survival (PFS) (A) and overall survival (OS) (B) of total population and stratified by sex*


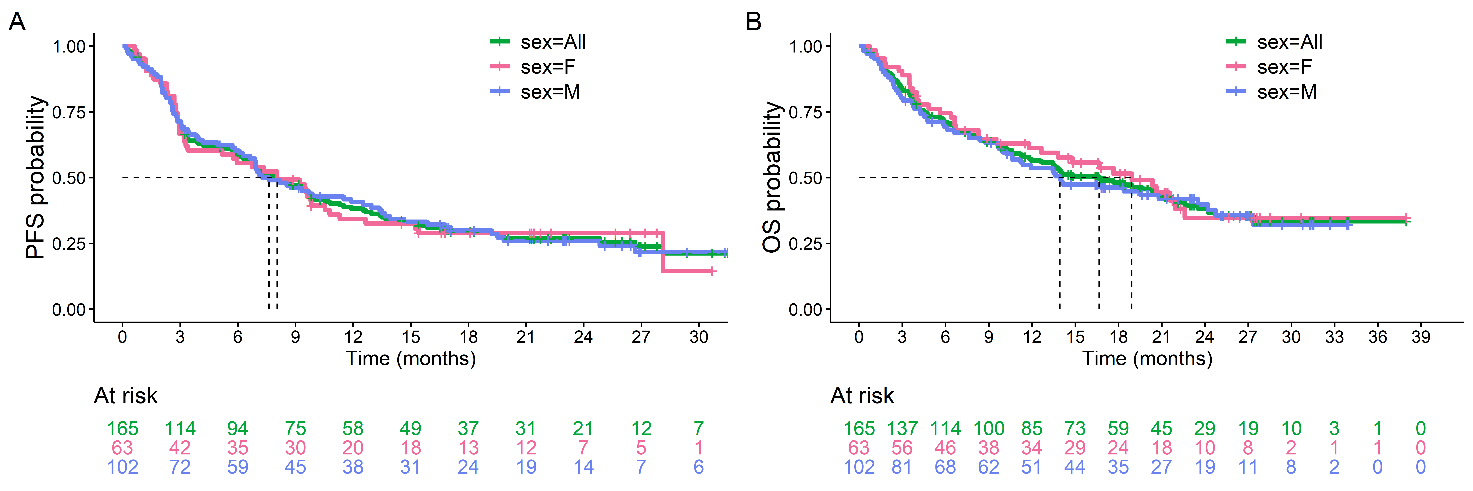


***Supplementary Figure 2****. Kaplan-Meier curves for progression free survival (PFS) of Melanoma patients (A) and NSCLC patients (B) of all population and stratified by sex*


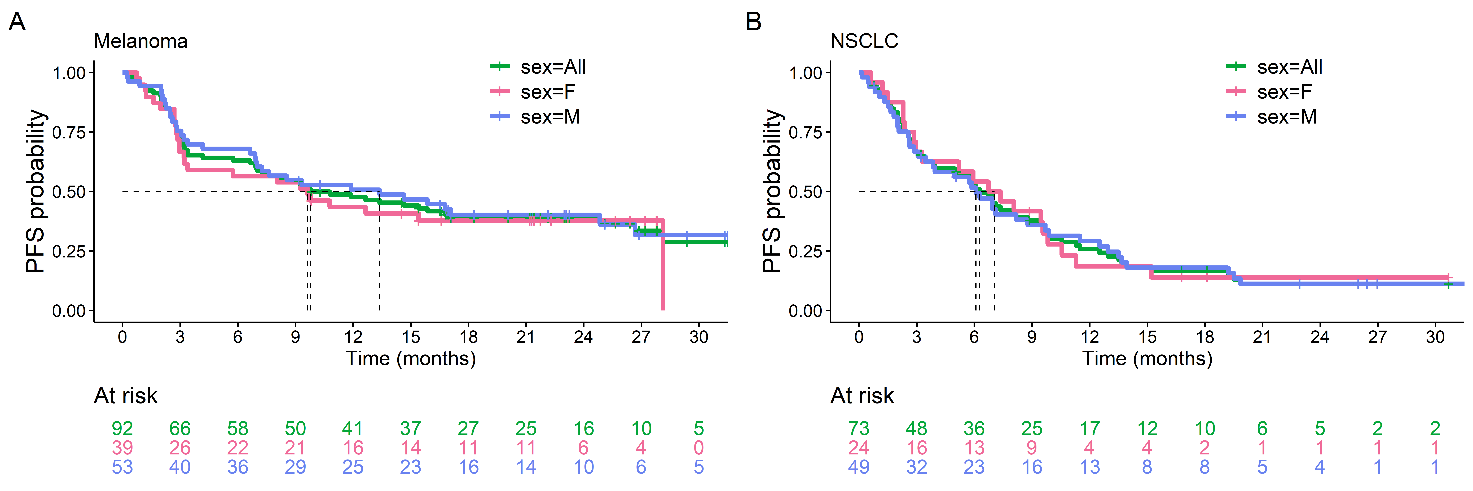


***Supplementary Figure 3****. Kaplan-Meier curves for overall survival (OS) of Melanoma patients (A) and NSCLC patients (B) of all population and stratified by sex*


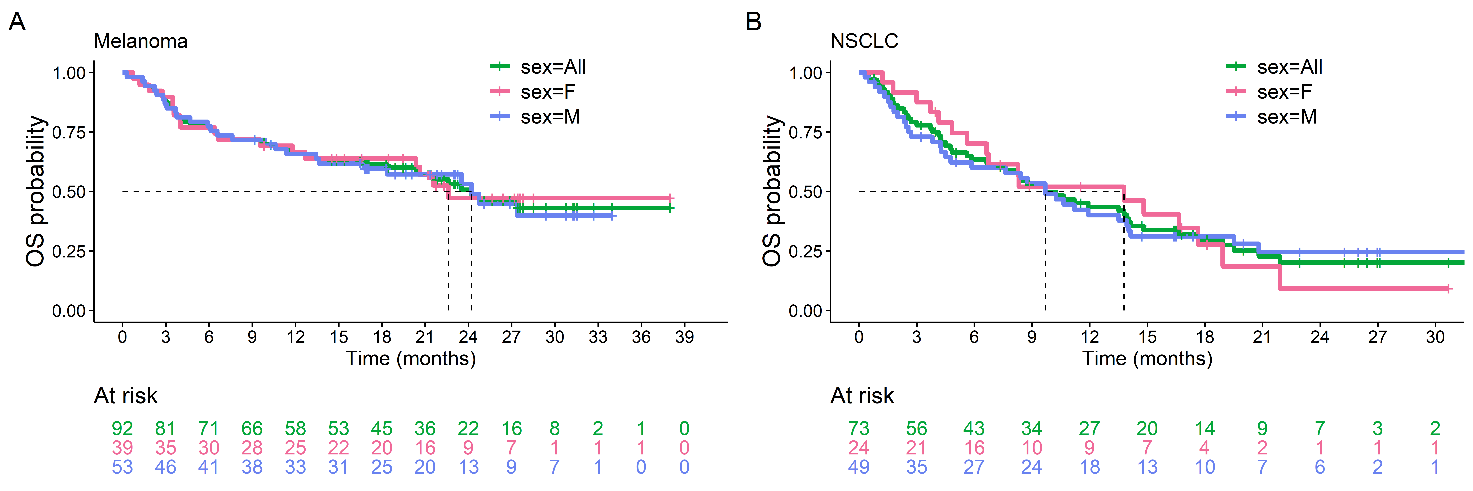


***Supplementary Figure 4****. Boxplot of circulating levels of biomarkers (pg/ml) in patients achieving disease control (CR/PR/SD) according to RECIST 1.1 criteria. Baseline values for the total population (A) and according to sex (B). Changes at cycle 2 from baseline values for the total population (C) and according to sex (D).*


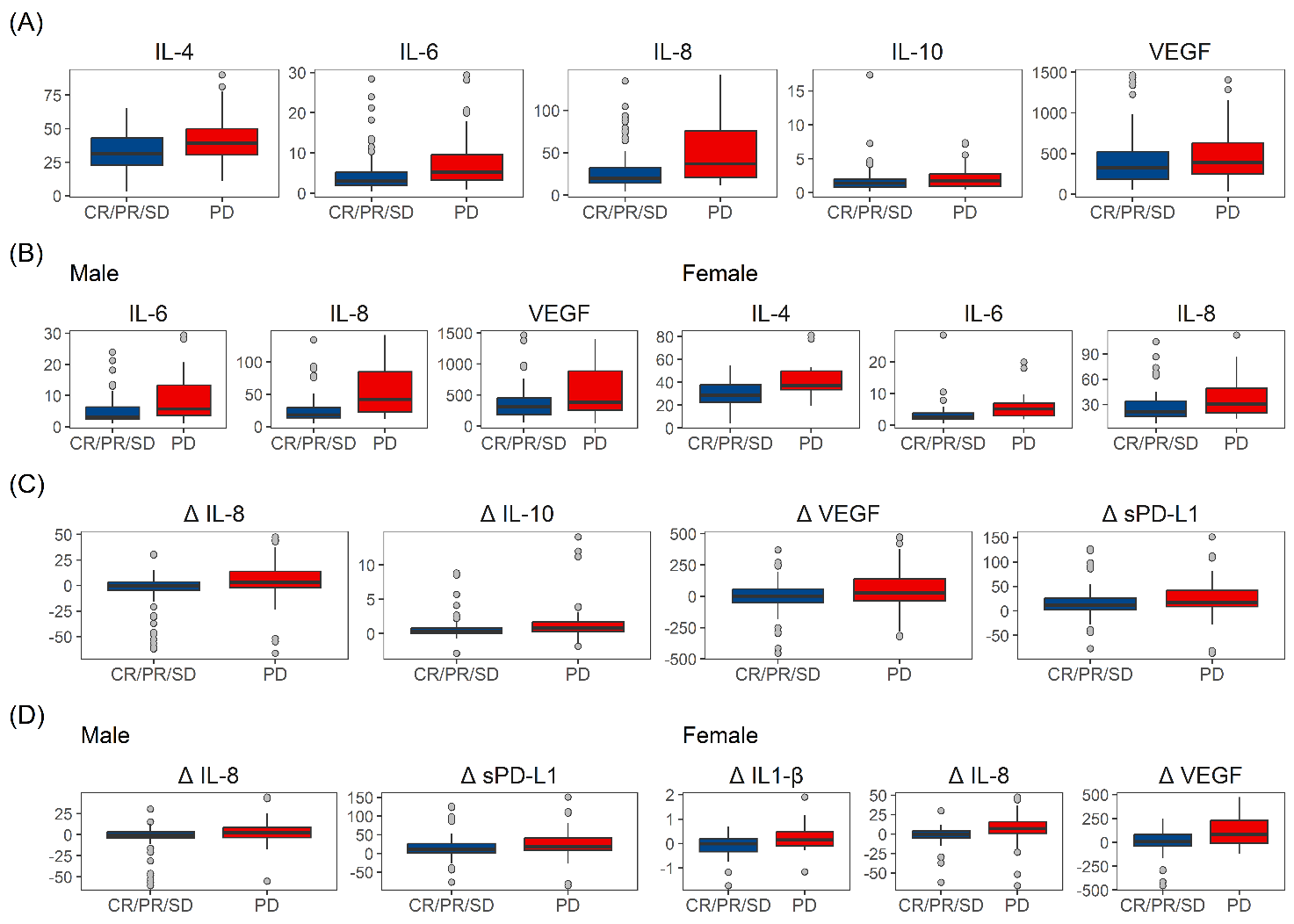


***Supplementary Table 1.*** *Italian Medicines Agency indications for Immune Checkpoint Inhibitors in Melanoma and NSCLC patients, at the time of study enrollment*

| **ICI** | **Dose regimen** | **Timing** | **Treatment Line** | **Association with chemo** | **PD-L1 (TPS)** |
| --- | --- | --- | --- | --- | --- |
| **Melanoma patients** |  |  |  |  |  |
| Nivolumab | 240 mg flat | Q2W | every | no | not required |
| Nivolumab | 480 mg flat | Q4W | every | no | not required |
| Pembrolizumab | 200 mg flat | Q3W | every | no | not required |
| **NSCLC patients** |  |  |  |  |  |
| Pembrolizumab | 200 mg flat | Q3W | 1st line | no | ≥50% |
|  | 200 mg flat | Q3W | 1st line | yes | 0-49% |
|  | 200 mg flat | Q3W | 2nd line | no | ≥1% |
| Atezolizumab | 1200 mg flat | Q3W | 1st line | no | ≥50% |
|  | 1200 mg flat | Q3W | 2nd line | no | ≥0% or ND |
| Nivolumab | 240 mg flat | Q2W | 2nd line | no | ≥0% or ND |
|  | 480 mg flat | Q4W | 2nd line | no | ≥0% or ND |

*Abbreviations:*

*Chemo, Chemotherapy; ICI, Immune Checkpoint Inhibitor; ND, Not Determined; NSCLC, Non-Small Cell Lung Cancer; PD-L1, Programmed Death - Ligand 1; Q2W, every 2 weeks; Q3W, every 3 weeks; Q4W, every 4 weeks; TPS, Tumor Proportion Score*

***Supplementary Table 2****. Patient characteristics for the melanoma study population*

| **Melanoma patients** |  | **Male (N=53)** | **Female (N=39)** | **Total (N=92)** | ***p value*** |
| --- | --- | --- | --- | --- | --- |
| **Age** | Median (Q1, Q3) | 73.0 (56.0, 78.0) | 67.0 (55.5, 80.0) | 71.0 (56.0, 78.2) | *0.6550* |
| **ECOG Performance Status** | N-Miss |  |  |  |  |
|  | 0 | 39 (73.6%) | 27 (69.2%) | 66 (71.7%) | *0.7060* |
|  | 1 | 12 (22.6%) | 9 (23.1%) | 21 (22.8%) |  |
|  | 2 | 2 (3.8%) | 3 (7.7%) | 5 (5.4%) |  |
| **Prior systemic treatment** | N-Miss |  | 1 | 1 |  |
|  | No | 48 (90.6%) | 32 (84.2%) | 80 (87.9%) | *0.3590* |
|  | Yes | 5 (9.4%) | 6 (15.8%) | 11 (12.1%) |  |
| **Current stage** | N-Miss |  |  |  |  |
|  | III | 8 (15.1%) | 6 (15.4%) | 14 (15.2%) | *0.9690* |
|  | IV | 45 (84.9%) | 33 (84.6%) | 78 (84.8%) |  |
| **Treatment** | Nivolumab | 41 (77.4%) | 31 (79.5%) | 72 (78.3%) | *0.8070* |
|  | Pembrolizumab | 12 (22.6%) | 8 (20.5%) | 20 (21.7%) |  |
| **RECIST** | CR | 8 (15.1%) | 4 (10.3%) | 12 (13.0%) | *0.4030* |
|  | PR | 19 (35.8%) | 9 (23.1%) | 28 (30.4%) |  |
|  | SD | 8 (15.1%) | 9 (23.1%) | 17 (18.5%) |  |
|  | PD | 18 (34.0%) | 17 (43.6%) | 35 (38.0%) |  |
| **Progression of disease** | No | 22 (41.5%) | 14 (35.9%) | 36 (39.1%) | *0.5860* |
|  | Yes | 31 (58.5%) | 25 (64.1%) | 56 (60.9%) |  |
| **Progression free survival (months)** | Median (95%CI) | 13.4 (6.9,26.7) | 9.6 (3.0,-) | 9.8 (6.9,17.1) | *0.6215* |
| **Status** | Alive | 27 (50.9%) | 21 (53.8%) | 48 (52.2%) | *0.7830* |
|  | Death | 26 (49.1%) | 18 (46.2%) | 44 (47.8%) |  |
| **Overall survival (months)** | Median (95%CI) | 24.2 (13.4,-) | 22.6 (11.8,-) | 24.2 (18.4,-) | *0.8458* |
| **Follow-up (months)** | Median (Q1, Q3) | 23.1 (18.9,31.3) | 22.3 (19.5,27.6) | 23.1 (18.9,28.5) |  |

***Supplementary Table 3.*** *Patient characteristics for the NSCLC study population*

| **NSCLC patients** |  | **Male (N=45)** | **Female (N=24)** | **Total (N=69)** | ***p value*** |
| --- | --- | --- | --- | --- | --- |
| **Age** | Median (Q1, Q3) | 72.0 (65.0, 76.0) | 67.0 (63.0, 71.5) | 70.0 (65.0, 75.0) | *0.0690* |
| **ECOG Performance Status** | N-Miss | 0 | 1 | 1 | *0.3010* |
|  | 0 | 13 (28.9%) | 6 (26.1%) | 19 (27.9%) |  |
|  | 1 | 28 (62.2%) | 17 (73.9%) | 45 (66.2%) |  |
|  | 2 | 4 (8.9%) | 0 (0.0%) | 4 (5.9%) |  |
| **Prior systemic treatment** | N-Miss |  |  |  |  |
|  | No | 36 (80.0%) | 19 (79.2%) | 55 (79.7%) | *0.9350* |
|  | Yes | 9 (20.0%) | 5 (20.8%) | 14 (20.3%) |  |
| **Current stage** | N-Miss | 2 |  | 2 |  |
|  | III | 3 (7.0%) | 1 (4.2%) | 4 (6.0%) | *0.6420* |
|  | IV | 40 (93.0%) | 23 (95.8%) | 63 (94.0%) |  |
| **Treatment** | Nivolumab | 5 (11.1%) | 2 (8.3%) | 7 (10.1%) | *0.5180* |
|  | Pembrolizumab | 18 (40.0%) | 14 (58.3%) | 32 (46.4%) |  |
|  | Atezolizumab | 4 (8.9%) | 2 (8.3%) | 6 (8.7%) |  |
|  | Chemo-immuno | 18 (40.0%) | 6 (25.0%) | 24 (34.8%) |  |
| **RECIST** | PR | 15 (33.3%) | 5 (20.8%) | 20 (29.0%) | 0.4470 |
|  | SD | 13 (28.9%) | 10 (41.7%) | 23 (33.3%) |  |
|  | PD | 17 (37.8%) | 9 (37.5%) | 26 (37.7%) |  |
| **Progression of disease** | No | 11 (24.4%) | 4 (16.7%) | 15 (21.7%) | *0.4560* |
|  | Yes | 34 (75.6%) | 20 (83.3%) | 54 (78.3%) |  |
| **Progression free survival (months)** | Median (95%CI) | 6.1 (3.1,8.8) | 7.1 (2.9,9.8) | 6.3 (3.8,8.8) | *0.9976* |
| **Status** | Alive | 14 (31.1%) | 7 (29.2%) | 21 (30.4%) | *0.8670* |
|  | Death | 31 (68.9%) | 17 (70.8%) | 48 (69.6%) |  |
| **Overall survival (months)** | Median (95%CI) | 9.7 (4.5,13.9) | 13.8 (5.6,17.6) | 9.7 (6.6,14.0) | *0.9607* |
| **Follow-up** | Median (Q1, Q3) | 22.9 (16.5,26.5) | 18.1 (11.5,--) | 22.9 (16.5,27.0) |  |

***Supplementary Table 4****. Baseline levels of biomarkers (pg/ml) according to sex*

| **Baseline marker levels** | **Male (N=98)** | **Female (N=63)** | **Total (N=161)** | ***p value*** |
| --- | --- | --- | --- | --- |
| MCP-1 | 432.5 (334.8, 540.8) | 454.0 (383.0, 546.0) | 448.0 (338.0, 545.0) | *0.5930* |
| IP-10 | 170.5 (116.5, 244.0) | 209.0 (132.5, 324.0) | 177.0 (120.0, 280.0) | *0.0820* |
| GM-CSF | 3.3 (2.0, 6.1) | 3.1 (1.9, 6.4) | 3.1 (1.9, 6.3) | *0.9100* |
| IL-1β | 1.0 (0.7, 1.7) | 1.0 (0.6, 1.4) | 1.0 (0.7, 1.6) | *0.5200* |
| IL-2 | 2.1 (1.4, 4.2) | 1.8 (1.3, 3.8) | 2.1 (1.4, 4.0) | *0.2940* |
| IL-4 | 37.6 (24.9, 46.4) | 35.2 (25.4, 45.0) | 35.7 (25.3, 45.7) | *0.6330* |
| IL-5 | 0.7 (0.4, 1.3) | 0.7 (0.4, 1.2) | 0.7 (0.4, 1.2) | *0.4920* |
| IL-6 | 4.3 (2.8, 10.0) | 3.3 (2.1, 5.5) | 3.9 (2.5, 7.8) | *0.0250* |
| IL-8 | 23.4 (15.2, 50.9) | 25.6 (18.1, 44.9) | 24.0 (15.9, 46.8) | *0.4110* |
| IL-10 | 1.6 (1.0, 2.2) | 1.4 (0.8, 2.0) | 1.5 (0.9, 2.2) | *0.4130* |
| TNF-α | 19.0 (15.7, 22.1) | 17.7 (15.3, 21.3) | 18.0 (15.7, 21.9) | *0.3660* |
| VEGF | 328.5 (217.2, 545.0) | 427.0 (247.5, 599.0) | 371.0 (225.0, 578.0) | *0.3000* |
| sPD-L1 | 97.1 (82.0, 118.5) | 92.6 (75.3, 111.0) | 94.5 (78.9, 117.0) | *0.2100* |

***Supplementary Table 5****. Baseline levels of biomarkers (pg/ml) according to diagnosis*

| **Baseline marker levels** | **Melanoma (N=92)** | **NSCLC (N=69)** | **Total (N=161)** | ***p value*** |
| --- | --- | --- | --- | --- |
| MCP-1 | 443.5 (362.5, 524.0) | 454.0 (327.0, 590.0) | 448.0 (338.0, 545.0) | *0.7570* |
| IP-10 | 173.0 (121.0, 280.0) | 188.0 (120.0, 279.0) | 177.0 (120.0, 280.0) | *0.8640* |
| GM-CSF | 2.9 (1.9, 6.0) | 3.8 (1.8, 6.5) | 3.1 (1.9, 6.3) | *0.2990* |
| IL-1β | 1.0 (0.7, 1.5) | 1.1 (0.6, 1.7) | 1.0 (0.7, 1.6) | *0.3030* |
| IL-2 | 2.1 (1.4, 3.7) | 2.2 (1.3, 4.0) | 2.1 (1.4, 4.0) | *0.5340* |
| IL-4 | 35.9 (26.1, 45.2) | 35.7 (25.2, 46.6) | 35.7 (25.3, 45.7) | *0.8750* |
| IL-5 | 0.8 (0.5, 1.3) | 0.7 (0.4, 1.2) | 0.7 (0.4, 1.2) | *0.5940* |
| IL-6 | 3.0 (2.0, 5.0) | 6.2 (3.2, 13.0) | 3.9 (2.5, 7.8) | *< 0.001* |
| IL-8 | 20.9 (15.4, 38.9) | 29.9 (19.7, 57.8) | 24.0 (15.9, 46.8) | *0.0190* |
| IL-10 | 1.5 (1.0, 2.0) | 1.5 (0.8, 2.6) | 1.5 (0.9, 2.2) | *0.4610* |
| TNF-α | 17.6 (15.4, 21.4) | 19.0 (15.8, 22.5) | 18.0 (15.7, 21.9) | *0.2930* |
| VEGF | 332.0 (197.0, 558.2) | 396.0 (239.0, 674.0) | 371.0 (225.0, 578.0) | *0.1740* |
| sPD-L1 | 89.4 (74.9, 111.8) | 104.0 (85.3, 123.0) | 94.5 (78.9, 117.0) | *0.0060* |

***Supplementary Table 6.*** *Multiple logistic regression predicting the objective response rate for biomarkers baseline levels (pg/ml) (A), and variation between cycle 2 and baseline (B)*

| **A** | **Sex** | **Baseline marker levels** | **cut-off** |  | **OR (95%CI)** | ***P value*** | ***P value interaction*** |
| --- | --- | --- | --- | --- | --- | --- | --- |
|  |  | IL-6 | 5.29 | low | Ref |  |  |
|  |  |  |  | high | 0.37 (0.17,0.81) | *0.0134* |  |
|  | M | TNF-α | 19.75 | low | Ref |  | *0.0386* |
|  |  |  |  | high | 1.57 (0.66,3.77) | *0.3111* |  |
|  | F | TNF-α |  | low | Ref |  |  |
|  |  |  |  | high | 0.23 (0.05,1.16) | *0.0747* |  |
| **B** |  | **Δ of cycle 2 marker levels from baseline** | **cut-off** |  | **OR (95%CI)** | ***P value*** |  |
|  |  | IL-8 | 3.23 | decrease | ref |  |  |
|  |  |  |  | increase | 0.36 (0.17,0.77) | *0.0086* |  |
|  |  | VEGF | 45.00 | decrease | ref |  |  |
|  |  |  |  | increase | 0.32 (0.15,0.69) | *0.0040* |  |

***Supplementary Table 7****. Multiple logistic regression predicting disease control rate of biomarkers baseline levels (pg/ml) (A), and variation between cycle 2 and baseline (B)*

| **A** | **Sex** | **Baseline marker levels** | **cut-off** |  | **OR (95%CI)** | ***P value*** |
| --- | --- | --- | --- | --- | --- | --- |
|  |  | IL-6 | 3.04 | low | Ref |  |
|  |  |  |  | high | 0.30 [0.14;0.66] | *0.0026* |
|  |  | IL-8 | 19.86 | low | Ref |  |
|  |  |  |  | high | 0.30 [0.14;0.66] | *0.0026* |
| **B** |  | **Δ of cycle 2 marker levels from baseline** | **cut-off** |  | **OR (95%CI)** | ***P value*** |
|  |  | sPD-L1 | 6.4 | decrease | Ref |  |
|  |  |  |  | increase | 0.40 [0.19,0.85] | *0.0180* |

***Supplementary Table 8****. Multiple Cox regression predicting the progression free survival (PFS) for biomarkers baseline levels (pg/ml) (A), and variation between cycle 2 and baseline (B)*

| **A** | **Sex** | **Baseline marker levels** | **cut-off** |  | **PFS HR (95%CI)** | ***P value*** | ***P value interaction*** |
| --- | --- | --- | --- | --- | --- | --- | --- |
|  |  | MCP-1 | 365 | low | Ref |  |  |
|  |  |  |  | high | 0.43 (0.28,0.66) | *0.0001* |  |
|  |  | IL-5 | 1.07 | low | Ref |  |  |
|  |  |  |  | high | 0.39 (0.25,0.60) | *0.0001* |  |
|  |  | IL-8 | 34.1 | low | Ref |  |  |
|  |  |  |  | high | 2.93 (1.77,4.84) | *0.0001* |  |
|  | M | IL-4 | 28.6 | low | Ref |  | *0.0002* |
|  |  |  |  | high | 0.98 (0.59,1.62) | *0.9339* |  |
|  | F | IL-4 |  | low | Ref |  |  |
|  |  |  |  | high | 7.35 (2.95,18.28) | *0.0001* |  |
|  | M | IL-6 | 2.97 | low | Ref |  | *0.0067* |
|  |  |  |  | high | 4.45 (2.35,8.44) | *0.0001* |  |
|  | F | IL-6 |  | low | Ref |  |  |
|  |  |  |  | high | 1.03 (0.45,2.36) | *0.9515* |  |
|  | M | IL-10 | 1.43 | low | Ref |  | *0.0239* |
|  |  |  |  | high | 0.92 (0.55,1.55) | *0.7635* |  |
|  | F | IL-10 |  | low | Ref |  |  |
|  |  |  |  | high | 2.62 (1.24,5.55) | *0.0115* |  |
|  | M | sPD-L1 | 111 | low | Ref |  | *0.0022* |
|  |  |  |  | high | 0.69 (0.39,1.22) | *0.2063* |  |
|  | F | sPD-L1 |  | low | Ref |  |  |
|  |  |  |  | high | 3.12 (1.37,7.13) | *0.0070* |  |
| **B** | **Sex** | **Δ of cycle 2 marker levels from baseline** | **cut-off** |  | **PFS HR (95%CI)** | ***P value*** |  |
|  |  | IL-8 | 4.63 | decrease | Ref |  |  |
|  |  |  |  | increase | 2.18 (1.44,3.31) | *0.0001* |  |
|  |  | TNF-α | 2.92 | decrease | Ref |  |  |
|  |  |  |  | increase | 0.59 (0.38,0.90) | *0.0144* |  |
|  |  | VEGF | 69 | decrease | ref |  |  |
|  |  |  |  | increase | 2.43 (1.63,3.62) | *0.0001* |  |

***Supplementary Table 9****. Multiple Cox regression predicting the overall survival (OS) for biomarkers baseline levels (pg/ml) (A), and variation between cycle 2 and baseline (B)*

| **A** | **Sex** | **Baseline marker levels** | **cut-off** |  | **OS HR (95%CI)** | ***P value*** | ***P value interaction*** |
| --- | --- | --- | --- | --- | --- | --- | --- |
|  |  | MCP-1 | 365 | low | Ref |  |  |
|  |  |  |  | high | 0.52 (0.34,0.82) | *0.0043* |  |
|  |  | IL-6 | 2.9 | low | Ref |  |  |
|  |  |  |  | high | 4.35 (2.55,7.41) | *0.0001* |  |
|  |  | IL-8 | 34.12 | low | Ref |  |  |
|  |  |  |  | high | 3.13 (1.99,4.94) | *0.0001* |  |
|  | M | GM-CSF | 4.03 | low | Ref |  | *0.0139* |
|  |  |  |  | high | 0.24 (0.12,0.47) | *0.0001* |  |
|  | F | GM-CSF |  | low | Ref |  |  |
|  |  |  |  | high | 0.81 (0.40,1.63) | *0.5598* |  |
|  | M | IL-1β | 1.22 | low | Ref |  | *0.0013* |
|  |  |  |  | high | 2.57 (1.37,4.82) | *0.0032* |  |
|  | F | IL-1β |  | low | Ref |  |  |
|  |  |  |  | high | 0.53 (0.25,1.11) | *0.0918* |  |
| **B** | **Sex** | **Δ of cycle 2 marker levels from baseline** | **cut-off** |  | **OS HR (95%CI)** | ***P value*** |  |
|  |  | IL-4 | 0.88 | decrease | Ref |  |  |
|  |  |  |  | increase | 1.92 (1.21,3.06) | *0.0060* |  |
|  |  | IL-5 | 0.06 | decrease | Ref |  |  |
|  |  |  |  | increase | 0.47 (0.30,0.76) | *0.0018* |  |
|  |  | IL-8 | 4.63 | decrease | Ref |  |  |
|  |  |  |  | increase | 2.24 (1.43,3.52) | *0.0001* |  |
|  |  | VEGF | 69 | decrease | Ref |  |  |
|  |  |  |  | increase | 2.20 (1.40,3.46) | *0.0001* |  |
